# Supplementary material for: Graphical Model Selection for Gaussian Conditional Random Fields in the Presence of Latent Variables
Source: J Am Stat Assoc. 2018 Jul 11;114(526):723–34. doi: 10.1080/01621459.2018.1434531 (PMC6636895; doi:10.1080/01621459.2018.1434531)
Supplement: Supplementary file 1 — Supplementary Materials [file UASA_A_1434531_SM8398.zip › UASA_A_1434531_Supplement.pdf]

Graphical Model Selection for Gaussian Conditional Random Fields in the Presence of Latent Variables: Supplementary Materials

Benjamin Frot*
Department of Statistics, University of Oxford
and
Luke Jostins
Wellcome Trust for Human Genetics, University of Oxford
and
Gilean McVean
Department of Statistics, University Of Oxford

November 12, 2017

*BF gratefully acknowledges the EPSRC and Amazon Web Services. LJ is supported by a Wellcome Trust grant (098759/Z/12/Z) and Christ Church, Oxford. GM is funded by the Wellcome Trust grant 100956/Z/13/Z. This work was jointly supervised by LJ and GM.

Contents

1 Introduction **3**

2 Theoretical Results: Supplementary Materials for Section 3 **5**

2.1 Derivation of the marginal log-likelihood 5

2.2 Identifiability 6

2.2.1 Key Definitions 7

2.2.2 Conditions on the Fisher Information Matrix (FIM) 8

2.2.3 An important result from the literature 10

2.3 Elementary properties of the likelihood 11

2.4 Curvature of the likelihood and the rank variety 13

2.4.1 Curvature of the rank variety 13

2.4.2 Curvature of the rank variety – Proof of Proposition 2 16

2.4.3 Curvature of the likelihood 17

2.5 Consistency 21

2.5.1 Statement of the result 22

2.5.2 Tangent space constraints 24

2.5.3 Variety constraints 29

2.5.4 From variety to tangent space constraints 36

2.5.5 From tangent space constraints to problem (2.2) 37

2.5.6 Bounding the error terms 37

2.5.7 Proof of consistency 42

2.6 A note on the validity of these results for arbitrary matrices 45

3 Optimisation: Supplementary Materials for Section 4 **46**

3.1 A three-block Alternative Direction Method of Multipliers (ADMM) 47
3.1.1 The Alternative Direction Method of Multipliers 47
3.1.2 Writing the updates of ADMM for Problem (3.1) 49
3.1.3 Computation of the proximal operator (3.6) 52
3.1.4 Summary 53
3.2 Semi-Definite Programming 54

4 Simulations: Supplementary Materials for Section 5 56
4.1 Description of the graphical models 56
4.2 Additional results 57

5 Application: Supplementary Materials for Section 6 61
5.1 Data Preparation 61
5.2 Enrichment Statistic 65
5.3 Results for SCGGM 66
5.4 Supplementary Figures 70

6 A Summary of our Notations 75

1 Introduction

The aim of this document is to complement *Graphical Model Selection for Gaussian Conditional Random Fields in the Presence of Latent Variables* with additional proofs and figures.

The log-likelihood

$$\ell(S_X, L_X, S_{ZX}, L_{ZX}; \Sigma_Z^n, \Sigma_X^n, \Sigma_{ZX}^n) = \log\det\left(S_X - L_X\right) - Tr\left(\Sigma_X^n(S_X - L_X)\right) - \\ 2Tr\left(\Sigma_{ZX}^n(S_{ZX} - L_{ZX})^T\right) - Tr\left(((S_X - L_X)^{-1}(S_{ZX} - L_{ZX})^T\Sigma_Z^n(S_{ZX} - L_{ZX})\right). \quad (1.1)$$

defined in Section 2 of the main paper will be studied in detail. It is clear that any choice of S_X, L_X and S_{ZX}, L_{ZX} that keeps the differences $K_X := S_X - L_X$, $K_{ZX} := S_{ZX} - L_{ZX}$ constant also keeps the log-likelihood constant. We will therefore often use a more convenient notation for the log-likelihood:

$$\ell(K_{ZX}, K_X; \Sigma_O^n) := -\log\det K_X + Tr(\Sigma_X^n K_X + 2K_{ZX}^T\Sigma_{ZX}^n + K_X^{-1}K_{ZX}^T\Sigma_Z^n K_{ZX}), \quad (1.2)$$

where Σ_O^n is shorthand for $\Sigma_Z^n, \Sigma_X^n, \Sigma_{ZX}^n$ and O indexes observed variables: $O := Z \cup X$. Following the notations introduced in Section 2, the superscript $\cdot^*$ will always denote the "true" value of the parameters. Thus, under our main assumption that

$$Y_X | Y_Z \sim \mathcal{N}\left\{-\left(S_X^* - L_X^*\right)^{-1}\left({S_{ZX}^*}^T - {L_{ZX}^*}^T\right)Y_Z, \left(S_X^* - L_X^*\right)^{-1}\right\}, \quad (1.3)$$

the log-likelihood evaluated at the true parameters is

$$\ell(K_X^*, K_{ZX}^*; \Sigma_O^n) = \ell(S_X^*, L_X^*, S_{ZX}^*, L_{ZX}^*; \Sigma_Z^n, \Sigma_X^n, \Sigma_{ZX}^n).$$

The rest of the document is organised as follows. In the next section, we prove a few elementary properties of the log-likelihood, recall results from the literature regarding identifiability and provide a proof of our consistency theorem (Theorem 1 in the main paper). We then show how either the alternative direction method of multipliers or semi-definite programming techniques can be used to compute estimates. Finally, we provide additional results and figures obtained on both simulated and real data. The last section contains a list of our notations.

2 Theoretical Results: Supplementary Materials for Section 3

Our goal is to provide a proof of Theorem 1 (main paper). In the first few subsections, we recall important results from the literature and extend existing ones to the case of non-square matrices (see Section 2.6 for more explanations about non-square matrices). The last subsection builds on these preliminary results and concludes the proof. The strategy we follow here is similar to the one developed in Chandrasekaran et al. (2012), which is itself reminiscent of the approach originally used in Wainwright (2009). However, almost all of our results differ due to the form taken by the likelihood. An overview of the proof technique is given in Subsection 2.5.

Throughout, we use notations that are consistent with the ones introduced in the main paper.

2.1 Derivation of the marginal log-likelihood

We now revisit the claim made in Section 2.1 (main paper) about the form taken by the marginal distribution (1.3). We take as a starting point our assumption about the joint distribution of Y_Z and Y_X:

$$\begin{pmatrix} Y_X \\ Y_H \end{pmatrix} | Y_Z \sim \mathcal{N} \left\{ - \begin{pmatrix} M_X^* & M_{XH}^* \\ {M_{XH}^*}^T & M_H^* \end{pmatrix}^{-1} \begin{pmatrix} {M_{ZX}^*}^T \\ {M_{ZH}^*}^T \end{pmatrix} Y_Z, \begin{pmatrix} M_X^* & M_{XH}^* \\ {M_{XH}^*}^T & M_H^* \end{pmatrix}^{-1} \right\},$$

which is of the form

$$\begin{pmatrix} Y_X \\ Y_H \end{pmatrix} | Y_Z \sim \mathcal{N} \left\{ -M^{*-1} \begin{pmatrix} {M_{ZX}^*}^T \\ {M_{ZH}^*}^T \end{pmatrix} Y_Z, M^{*-1} \right\}.$$

After marginalisation over Y_H, the conditional mean $Y_X|Y_Z$ is given by the upper-part of the conditional mean vector $-M^{*-1}\begin{pmatrix} {M^*_{ZX}}^T \\ {M^*_{ZH}}^T \end{pmatrix} Y_Z$. The marginal covariance matrix is given by extracting the upper-left block of M^{*-1}, *i.e.* $(M^{*-1})_X$. To express these quantities in terms of the nominal parameters M^*_X, M^*_{ZX}, etc..., we can use the formula for the inverse of a partitioned matrix:

$$M^{*-1} = \begin{pmatrix} \left(M^*_X - M^*_{XH} M^{*-1}_H M^*_{HX}\right)^{-1} & -\left(M^*_X - M^*_{XH} M^{*-1}_H M^*_{HX}\right)^{-1} M^*_{XH} M^{*-1}_H \\ \cdot & \left(M^*_H - M^*_{HX} M^{*-1}_X M^*_{XH}\right)^{-1} \end{pmatrix}.$$

It follows that the marginal covariance matrix is given by

$$(M^{*-1})_X = \left(M^*_X - M^*_{XH} M^{*-1}_H M^*_{HX}\right)^{-1}.$$

The conditional mean vector for Y_X can also be rewritten:

$$-\left(\left(M^*_X - M^*_{XH} M^{*-1}_H M^*_{HX}\right)^{-1} {M^*_{ZX}}^T - \left(M^*_X - M^*_{XH} M^{*-1}_H M^*_{HX}\right)^{-1} M^*_{XH} M^{*-1}_H {M^*_{ZH}}^T\right) Y_Z,$$

which factorises as

$$-\left(M^*_X - M^*_{XH} M^{*-1}_H M^*_{HX}\right)^{-1} \left({M^*_{ZX}}^T - M^*_{XH} M^{*-1}_H {M^*_{ZH}}^T\right) Y_Z.$$

As in Section 2 of the main paper, we define $S^*_X := M^*_X$, $L^*_X := M^*_{XH} M^{*-1}_H {M^*_{XH}}^T$, $S^*_{ZX} := M^*_{ZX}$ and $L^*_{ZX} := M^*_{XH} M^{*-1}_H {M^*_{ZH}}^T$. This yields expression (1.3).

2.2 Identifiability

As already mentioned in Section 3 of the main paper, the question of identifiability has already been answered in Chandrasekaran et al. (2012). The reason for this is that only a number of properties of the Fisher Information Matrix (FIM) appear in the proofs but the

actual form of the FIM is irrelevant. Here, we start by recalling important definitions from the literature before stating a proposition which is instrumental in the proof of consistency. In what follows we will refer to this proposition time and again. More details about the motivation behind these definitions are given in Chandrasekaran et al. (2009).

2.2.1 Key Definitions

We write $\mathcal{S}(k)$ for the algebraic variety of $(m+p)\times p$ matrices with at most k non-zero entries. Any $(m+p)\times p$ matrix M with support of cardinality k is a smooth point of $\mathcal{S}(k)$ and we denote by $\Omega(M)$ the tangent space to $\mathcal{S}(k)$ at M (this is the set of matrices in $\mathcal{S}(k)$ whose support is included in the support of M).

Similarly, we define $\mathcal{L}(r)$, the variety of $(m+p)\times p$ matrices with rank at most r. Any matrix M of rank r is a smooth point of $\mathcal{L}(r)$ and we write $T(M)$ for the tangent space to $\mathcal{L}(r)$ at M (this is the set of matrices in $\mathcal{L}(r)$ whose row space is identical to M's or whose column space is identical to M's).

Given two linear spaces T_1, T_2 of identical dimensions, write

$$\rho(T_1,T_2) \triangleq \max_{\|N\|_2\le 1} \|(\mathcal{P}_{T_1}-\mathcal{P}_{T_2})(N)\|_2 \tag{2.1}$$

for a measure of the "angle" between T_1 and T_2. Here $\mathcal{P}_{T_1}$ is the orthogonal projector onto T_1 while $\|N\|_2$ is the spectral norm of N (*i.e.* its largest singular value). $\rho(T_1,T_2)$ is used to describe the set of tangent spaces that are close to the nominal $T(L^*)$.

Chandrasekaran et al. (2009) quantify the spread of the effect of the latent variables on the observed variables by defining

$$\xi(T(M)) \triangleq \max_{N\in T(M),\|N\|_2\le 1} \|N\|_\infty$$

for any matrix M. Remark that $\xi(T(M))$ controls all the elements of the tangent space at M. Thus, if $\xi(T(M))$ is small, none of the matrices having the same row-space (resp.

column-space) as M can have a large ℓ_∞-norm, meaning that the row-space (resp. column-space) of M cannot be closely aligned with any of the coordinate axes. Therefore, a small $\xi(T(M))$ guarantees that no single latent variable will have a strong effect on only a small set of the observed variables (precisely because it is the observed variables that define the coordinate axes).

Similarly, define

$$\mu(\Omega(M)) \triangleq \max_{N\in\Omega(M),\|N\|_\infty\leq 1} \|N\|_2 \,,$$

to quantify the diffusivity of the spectrum of M is. One shows that matrices with a small number of non-zero entries per row/column (and thus sparse) have a small $\mu(M)$. In particular, $\mu(\Omega(M)) \leq \text{degree}(M)$.

2.2.2 Conditions on the Fisher Information Matrix (FIM)

In the context of the graphical lasso, the *mutual incoherence* or *irrepresentability* condition is a well-known requirement for identifiability (Ravikumar et al. 2011). Just like the irrepresentability condition imposes restrictions on the Hessian of the likelihood, the conditions given in Chandrasekaran et al. (2012) involve the Fisher Information Matrix (FIM) $\mathcal{I}^*_{\Sigma_Z^n}$ evaluated at the true parameters $S^* - L^*$. The subscript is a reminder that the entire analysis is performed conditional on Σ_Z^n. To avoid cluttered notations, we write $\Omega = \Omega(S^*)$ and $T = T(L^*)$ and denote by $\mathcal{P}_\Omega, \mathcal{P}_T$ the orthogonal projections onto these linear subspaces.

We can now define the quantities that control the behaviour of the FIM when restricted to Ω and T. Set

$$\begin{aligned}
\alpha_\Omega &\triangleq \min_{M\in\Omega,||M||_\infty=1} ||\mathcal{P}_\Omega \mathcal{I}^*_{\Sigma_Z^n} \mathcal{P}_\Omega(M)||_\infty; \\
\delta_\Omega &\triangleq \max_{M\in\Omega,||M||_\infty=1} ||\mathcal{P}_{\Omega^\perp} \mathcal{I}^*_{\Sigma_Z^n} \mathcal{P}_\Omega(M)||_\infty, \\
\beta_\Omega &\triangleq \max_{M\in\Omega,||M||_2=1} ||\mathcal{I}^*_{\Sigma_Z^n}(M)||_2.
\end{aligned}$$

Likewise let,

$$\alpha_T \triangleq \min_{\rho(T,T')<\xi(T)/2} \min_{M\in T', ||M||_2=1} ||\mathcal{P}_{T'}\mathcal{I}^*_{\Sigma_Z^n}\mathcal{P}_{T'}(M)||_2;$$
$$\delta_T \triangleq \max_{\rho(T,T')<\xi(T)/2} \max_{M\in T', ||M||_2=1} ||\mathcal{P}_{T'^\perp}\mathcal{I}^*_{\Sigma_Z^n}\mathcal{P}_{T'}(M)||_2,$$
$$\beta_T \triangleq \max_{\rho(T,T')<\xi(T)/2} \max_{M\in T', ||M||_\infty=1} ||\mathcal{I}^*_{\Sigma_Z^n}(M)||_\infty.$$

Finally, we define

$$\alpha \triangleq \min(\alpha_\Omega, \alpha_T) \quad \beta \triangleq \max(\beta_\Omega, \beta_T) \quad \delta \triangleq \max(\delta_\Omega, \delta_T).$$

Then the following assumption is a generalisation of the irrepresentability condition, and from now on we assume that it holds.

Assumption 1. *(Generalised Irrepresentability Condition, Chandrasekaran et al. (2012). Called Assumption 2 in the main paper.)*
There exists a $\nu \in (0, \frac{1}{2}]$ *such that*

$$\frac{\delta}{\alpha} \leq 1 - 2\nu.$$

A direct consequence of Assumption (1) is that $\alpha > 0$, so that the FIM is injective on Ω and on all the spaces that are close to T (including T itself). For that reason, Assumption (1) subsumes the standard *restricted convexity assumption* which is also necessary in the context of sparse conditional Gaussian Markov random fields and regularised regression in order to guarantee that the likelihood – when restricted to the true support – is strictly convex, and therefore that there exists a unique optimum (Wytock & Kolter 2013, Wainwright 2009).

Another consequence of Assumption 1 is Proposition 1 given in Section 2.2.3. This proposition is applied multiple times throughout the consistency proof. It holds only if the following conditions are met. This shows the role played by μ and ξ in identifiability.

Assumption 2. *(Assumptions for Proposition 1. Called Assumption 1 in the main paper.)*

$$\mu(\Omega)\xi(T) \leq \frac{1}{6}\left(\frac{\nu\alpha}{\beta(2-\nu)}\right)^2$$

and γ is chosen in the range

$$\gamma \in \left[\frac{3\xi(T)\beta(2-\nu)}{\nu\alpha}, \frac{\nu\alpha}{2\mu(\Omega)\beta(2-\nu)}\right].$$

Unsurprisingly, a sparse S^* (small $\mu(\Omega)$) and a diffuse effect of the latent variables on the observed nodes (small $\xi(T)$) increase the chances that Assumption 2 will hold.

2.2.3 An important result from the literature

We now recall a proposition of Chandrasekaran et al. (2012) which is instrumental in the consistency proof and to which we will refer time and again.

We first introduce a few additional notations. First, let g_γ denote the dual norm of the regularisation function $f_\gamma = \lambda(\gamma \|S\|_1 + \|L\|_*)$:

$$g_\gamma = \max\left(\frac{\|S\|_\infty}{\gamma}, \|L\|_2\right).$$

We also write $\mathcal{A} : \mathbb{R}^{(m+p)\times p} \times \mathbb{R}^{(m+p)\times p} \rightarrow \mathbb{R}^{(m+p)\times p}$ for the operator that adds two matrices. As usual, $\mathcal{A}^\dagger$ denotes its adjoint : $\mathcal{A}^\dagger : M \mapsto (M, M)$, so that $\mathcal{A}^\dagger\mathcal{A}(A, B) = (A+B, A+B)$.

Then, under Assumptions 1 and 2 we have the following result.

Proposition 1. *(Chandrasekaran et al. (2012), Proposition 3.3)*
Let $\mathcal{Y} = \Omega \times T'$ with $\rho(T, T') \leq \frac{\xi(T)}{2}$ (Recall that $\rho, \alpha, etc...$ were defined above in 2.2.1 and 2.2.2). Then

1.

$$\min_{(S,L)\in\mathcal{Y}, \|S\|_\infty=\gamma, \|L\|_2=1} g_\gamma(\mathcal{P}_\mathcal{Y}\mathcal{A}^\dagger\mathcal{I}^*_{\Sigma^n_Z}\mathcal{A}\mathcal{P}_\mathcal{Y}(S, L)) \geq \frac{\alpha}{2}$$

and specifically, for $(S, L) \in \mathcal{Y}$

$$g_\gamma(\mathcal{P}_\mathcal{Y}\mathcal{A}^\dagger\mathcal{I}^*_{\Sigma^n_Z}\mathcal{A}\mathcal{P}_\mathcal{Y}(S,L)) \geq \frac{\alpha}{2} g_\gamma(S,L).$$

2. *Writing* $\mathcal{Y}^\perp$ *for the orthogonal complement of* $\mathcal{Y}$*, we have*

$$\left\| \mathcal{P}_{\mathcal{Y}^\perp}\mathcal{A}^\dagger\mathcal{I}^*_{\Sigma^n_Z}\mathcal{A}\mathcal{P}_\mathcal{Y}(\mathcal{P}_\mathcal{Y}\mathcal{A}^\dagger\mathcal{I}^*_{\Sigma^n_Z}\mathcal{A}\mathcal{P}_\mathcal{Y})^{-1} \right\|_{g_\gamma \to g_\gamma} \leq 1 - \nu.$$

And, more specifically,

$$g_\gamma(\mathcal{P}_{\mathcal{Y}^\perp}\mathcal{A}^\dagger\mathcal{I}^*_{\Sigma^n_Z}\mathcal{A}\mathcal{P}_\mathcal{Y}(S,L)) \leq (1-\nu) g_\gamma(\mathcal{P}_\mathcal{Y}\mathcal{A}^\dagger\mathcal{I}^*_{\Sigma^n_Z}\mathcal{A}\mathcal{P}_\mathcal{Y}(S,L)).$$

2.3 Elementary properties of the likelihood

We now make a number of straightforward calculations about the likelihood

$$\ell(K_{ZX}, K_X; \Sigma^n_O) = -\log\det K_X + Tr(\Sigma^n_X K_X + 2K^T_{ZX}\Sigma^n_{ZX} + K_X^{-1}K^T_{ZX}\Sigma^n_Z K_{ZX}).$$

We are interested in computing the gradient and the Hessian of ℓ. In passing, we show that ℓ is *convex*, so that our objective function – being the sum of three convex functions – is also convex.

Property 1. *(Maximum Likelihood Estimate (M.L.E.))*
Assuming Σ^n_Z *is non-singular, the M.L.E. is given by*

$$\begin{aligned} \hat{K}_X &= (\Sigma^n_X - {\Sigma^n_{ZX}}^T {\Sigma^n_Z}^{-1}\Sigma^n_{ZX})^{-1}; \\ \hat{K}_{ZX} &= -{\Sigma^n_Z}^{-1}\Sigma^n_{ZX}\hat{K}_X. \end{aligned}$$

We can now compute the Hessian of ℓ.

Property 2. *(Fisher Information Matrix)*

$$\mathcal{I}^*_{\Sigma^n_Z} = -\begin{pmatrix} K_X^{*-1} \otimes K_X^{*-1} & 0 \\ 0 & 0 \end{pmatrix} - 2K_X^{*-1} \otimes \begin{pmatrix} K_X^{*-1}K^{*T}_{ZX}\Sigma^n_Z K^*_{ZX}K_X^{*-1} & -K_X^{*-1}K^{*T}_{ZX}\Sigma^n_Z \\ \cdot & \Sigma^n_Z \end{pmatrix}.$$

Proof. We use differentials.

First, it is easy to see that

$$\begin{aligned} d\ell &= -tr(K_X^{-1}dK_X) + tr(\Sigma_X^n dK_X) + 2tr(\Sigma_{ZX}^n (dK_{ZX})^T) \\ &\quad +2tr(\Sigma_Z^n dK_{ZX} K_X^{-1} K_{ZX}^T) - tr(\Sigma_Z^n K_{ZX} K_X^{-1} dK_X K_X^{-1} K_{ZX}^T). \end{aligned}$$

Likewise, it is straightforward to compute the second order derivatives. To make the computation easier to follow, we break down $d^2\ell$ into its individual components:

$$\begin{aligned} d(-tr(K_X^{-1}dK_X)) &= tr(K_X^{-1}dK_X A_X^{-1}dK_X); \\ d(tr(\Sigma_X^n dK_X)) &= 0; \\ d(tr(\Sigma_{ZX}^n (dK_{ZX})^T)) &= 0; \\ d(tr(\Sigma_Z^n dK_{ZX} K_X^{-1} K_{ZX}^T)) &= -tr(K_X^{-1}dK_X K_X^{-1} K_{ZX}^T \Sigma_Z^n dK_{ZX}) \\ &\quad +tr(K_X^{-1} dK_{ZX}^T \Sigma_Z^n dK_{ZX}); \\ d(-tr(\Sigma_Z^n K_{ZX} K_X^{-1} dK_X K_X^{-1} K_{ZX}^T)) &= 2tr(K_X^{-1}dK_X K_X^{-1} K_{ZX}^T \Sigma_Z^n K_{ZX} K_X^{-1} dK_X) \\ &\quad -2tr(K_X^{-1} dK_{ZX}^T \Sigma_Z^n K_{ZX} K_X^{-1} dK_X). \end{aligned}$$

So that:

$$\begin{aligned} d^2\ell &= tr(K_X^{-1}dK_X K_X^{-1}dK_X) + 2tr(K_X^{-1}dK_{ZX}^T \Sigma_Z^n dK_{ZX}) \\ &\quad +2tr(K_X^{-1}dK_X K_X^{-1} K_{ZX}^T \Sigma_Z^n K_{ZX} K_X^{-1} dK_X) \\ &\quad -4tr(K_X^{-1} dK_{ZX}^T \Sigma_Z^n K_{ZX} K_X^{-1} dK_X). \end{aligned}$$

Now, write $K := \begin{pmatrix} K_X \\ K_{ZX} \end{pmatrix}$ and similarly $vec(K) := \begin{pmatrix} vec(K_X) \\ vec(K_{ZX}) \end{pmatrix}$. Then, using the identity $tr(ABCD) = (vecB^T)^T (A^T \otimes C) vecD$, we obtain

$$\begin{aligned} tr(K_X^{-1}dK_X K_X^{-1}dK_X) &= d(vecK_X)^T (K_X^{-1} \otimes K_X^{-1}) dvecK_X \\ tr(K_X^{-1}dK_{ZX}^T \Sigma_Z^n dK_{ZX}) &= d(vecK_{ZX})^T (K_X^{-1} \otimes \Sigma_Z^n) dvecK_{ZX} \\ tr(K_X^{-1}dK_X K_X^{-1} K_{ZX}^T \Sigma_Z^n K_{ZX} K_X^{-1} dK_X) &= d(vecK_X)^T (K_X^{-1} \otimes K_X^{-1} K_{ZX}^T \Sigma_Z^n K_{ZX} K_X^{-1}) dvecK_X \\ tr(K_X^{-1}dK_{ZX}^T \Sigma_Z^n K_{ZX} K_X^{-1} dK_X) &= d(vecK_{ZX})^T (K_X^{-1} \otimes \Sigma_Z^n K_{ZX} K_X^{-1}) dvecK_X. \end{aligned}$$

So that,

$$\begin{aligned} d^2\ell &= d(vecK_X)^T \left[(K_X^{-1} \otimes K_X^{-1}) + 2(K_X^{-1} \otimes K_X^{-1}K_{ZX}^T\Sigma_Z^n K_{ZX}K_X^{-1})\right] dvecK_X \\ & \quad 2d(vecK_{ZX})^T(K_X^{-1} \otimes \Sigma_Z^n)dvecK_{ZX} \\ & \quad -4d(vecK_{ZX})^T(K_X^{-1} \otimes \Sigma_Z^n K_{ZX}K_X^{-1})dvecK_X \end{aligned}$$

and

$$d^2\ell = (dvecK)^T \begin{pmatrix} K_X^{-1} \otimes K_X^{-1} + 2K_X^{-1} \otimes K_X^{-1}K_{ZX}^T\Sigma_Z^n K_{ZX}K_X^{-1} & -2K_X^{-1} \otimes \Sigma_Z^n K_{ZX}K_X^{-1} \\ \cdot & 2K_X^{-1} \otimes \Sigma_Z^n \end{pmatrix} dvecK.$$

From which the result follows. □

In passing, remark that $-\mathcal{I}_{\Sigma_Z^n}(K)$ is positive semi-definite if and only if

$$\begin{pmatrix} K_X^{-1}K_{ZX}^T\Sigma_Z^n K_{ZX}K_X^{-1} & -K_X^{-1}K_{ZX}^T\Sigma_Z^n \\ \cdot & \Sigma_Z^n \end{pmatrix}$$

is positive semi-definite. This is because K_X is positive definite (we have the constraint $K_X \succ 0$). But it is easy to see that this matrix is of the form A^TA. In conclusion, ℓ is convex and so is the objective function.

2.4 Curvature of the likelihood and the rank variety

2.4.1 Curvature of the rank variety

Here, we extend two results of Chandrasekaran et al. (2012) from the special case of positive definite matrices to arbitrary matrices. We rely on previous work by Bach (2008).

We state here the results that we seek to prove and dedicate the rest of this section to their proof.

Given two linear subspaces $T1, T2$ of same dimension define

$$\rho(T1, T2) \triangleq \|\mathcal{P}_{T_1} - \mathcal{P}_{T_2}\|_{2\to 2} = \max_{\|N\|_2 \le 1} \|(\mathcal{P}_{T_1} - \mathcal{P}_{T_2})(N)\|_2 \,,$$

which measures the "angle" between these two subspaces. Recall that for any matrix $M \in \mathbb{R}^{p\times q}$, $T(M)$ denotes the tangent space to the variety of low-rank matrices at M. Then we have the following proposition.

Proposition 2. *(Extension of Chandrasekaran et al. (2012) (suppl. mat.), Proposition 2.1, 2.2.)*

Let $W \in \mathbb{R}^{p\times q}$ be a rank $r < min(p, q)$ matrix with non-zero singular values $(\sigma_i)_{i\in\{1,\dots,r\}}$. Write $\sigma = \min_i \sigma_i$, and let Δ be such that $||\Delta||_2 < \frac{\sigma}{4}$. Let $W + \Delta$ be a rank r matrix. Then we have

1. $$\rho(T(W + \Delta), T(W)) \le \frac{8}{\sigma}||\Delta||_2;$$

2. $$\left\|\mathcal{P}_{T(W)^\perp}(\Delta)\right\|_2 \le \frac{4}{\sigma}||\Delta||_2^2.$$

Preliminary Results: Jordan-Wielandt Matrix and Matrix perturbation bounds
Throughout, we assume that W is some $\mathbb{R}^{p\times q}$ matrix, with non-zero singular values $\sigma_i, i = 1, \dots, r$ (indexed by decreasing order) and singular vectors, u_i, v_i. The corresponding Jordan-Wielandt matrix is defined from W as follows (Stewart & Sun 1990):

$$\bar{W} = \begin{pmatrix} 0 & W \\ W^T & 0 \end{pmatrix}.$$

This matrix is interesting because it has eigenvalues $\sigma_i, -\sigma_i$ and its eigenvectors are $\frac{1}{\sqrt{2}}\begin{pmatrix} u_i \\ \pm v_i \end{pmatrix}$. We write $\bar{W} = \bar{U}\bar{S}\bar{U}^T$ for the eigenvalue decomposition of $\bar{W}$. If the singular value decomposition of W is $W = USV^T$, then we have $\bar{S} = \frac{1}{\sqrt{2}}\begin{pmatrix} S & 0 \\ 0 & -S \end{pmatrix}$ and $\bar{S} = \frac{1}{\sqrt{2}}\begin{pmatrix} U & U \\ V & -V \end{pmatrix}$ so that $\bar{U}\bar{U}^T = \begin{pmatrix} UU^T & 0 \\ 0 & VV^T \end{pmatrix}$.

Now, let Δ be a small perturbation to W. If $||\Delta||_2 \leq \frac{\sigma_r}{2}$ then $W + \Delta$ has r singular values that are strictly greater than $\sigma_r/2$ and all the remaining ones are strictly less than $\sigma_r/2$ (Stewart & Sun 1990). Let $P_{\bar{W},\sigma_r/2}$ denote the projector on the $p+q-2r$-dimensional invariant subspace of $\bar{W}$ which corresponds to the smallest eigenvalues (in this case it is a null space as W is of rank exactly r). Likewise, if $||\Delta||_2 \leq \sigma_r/2$, $P_{\bar{W}+\bar{\Delta},\sigma_r/2}$ also denotes a projector onto a $p + q - 2r$ dimensional subspace. The projection onto the orthogonal subspace is given by $I - P_{\bar{W}+\bar{\Delta},\sigma_r/2}$.

Bach (2008) shows the following results

Proposition 3. *(Bach (2008), Proposition 16)*
Assume W is of rank r and $||\Delta||_2 < \frac{\sigma_r}{4}$. Then, the projection on the first r eigenvectors of $\bar{W}$, $I - P_{\bar{W},\sigma_r/2}$, is such that

$$\left\|P_{\bar{W}+\bar{\Delta},\sigma_r/2} - P_{\bar{W},\sigma_r/2}\right\|_2 \leq \frac{4}{\sigma_r}||\Delta||_2.$$

Now, recall that for any two matrices A, B (of compatible dimensions) we have the following inequalities:

$$\max(||A||_2, ||B||_2) \leq \left\|\begin{pmatrix} A \\ B \end{pmatrix}\right\|_2 \leq ||A||_2 + ||B||_2.$$

Writing $P_{U(W)}$ (resp. $P_{V(W)}$) for the projection onto the row-space $U(W)$ (resp. $(V(M))$) then we have the following corollary

Corollary 1. *Assume W is of rank r and $||\Delta||_2 < \frac{\sigma_r}{4}$. Assume further that rank$(W+\Delta) = r$. Then we have that*

$$\max\left(\left\|P_{U(W+\Delta)} - P_{U(W)}\right\|_2, \left\|P_{V(W+\Delta)} - P_{V(W)}\right\|_2\right) \leq \left\|P_{\bar{W}+\bar{\Delta},\sigma_r/2} - P_{\bar{W},\sigma_r/2}\right\|_2.$$

We also have the following result from the literature:

Proposition 4. *(Bach (2008), Proposition 18)*
Assume that W has rank $r < \min(p,q)$, with singular value decomposition $W = USV^T$. If $\frac{4}{\sigma_r}||\Delta||_2^2 < ||(I - UU^T)\Delta(I - VV^T)||_2$, then rank$(W + \Delta) > r$.

2.4.2 Curvature of the rank variety – Proof of Proposition 2

Let W be a $p \times q$ matrix defined as before. The projection onto the matrix variety of low-rank matrices at W, $T(W)$, is written $\mathcal{P}_{T(W)}$. For any matrix N, it can be expressed using the row/column projectors as follows (Chandrasekaran et al. (2009)):

$$\mathcal{P}_{T(W)}(N) = P_{U(W)}N + NP_{V(W)} - P_{U(W)}NP_{V(W)},$$

while the projector onto the orthogonal subspace is $(I - \mathcal{P}_{T(W)})$, *i.e.*:

$$\mathcal{P}_{T(W)^{\perp}} = (I - P_{U(W)})N(I - P_{V(W)}).$$

For any matrix N we have that

$$\left(\mathcal{P}_{T(W+\Delta)} - \mathcal{P}_{T(W)}\right) = \left(P_{U(W+\Delta)} - P_{U(W)}\right) N \left(I - P_{V(W)}\right) + \left(I - P_{U(W+\Delta)}\right) N \left(P_{V(W+\Delta)} - P_{V(W)}\right).$$

As a result, under the assumptions of Proposition 2 (in particular that $||\Delta||_2 \leq \sigma_r/4$), we have the following inequalities:

$$
\begin{aligned}
\rho(T(W+\Delta),T(W)) \quad &\leq \quad \max_{||N||_2\leq 1} || \left(P_{U(W+\Delta)} - P_{U(W)}\right) N \left(I - P_{V(W)}\right) + \\
&\qquad \left(I - P_{U(W+\Delta)}\right) N \left(P_{V(W+\Delta)} - P_{V(W)}\right) ||_2 \\
&\leq \quad \max_{||N||_2\leq 1} \left\| \left(P_{U(W+\Delta)} - P_{U(W)}\right) N \left(I - P_{V(W)}\right)\right\|_2 + \\
&\qquad \max_{||N||_2\leq 1} \left\| \left(I - P_{U(W+\Delta)}\right) N \left(P_{V(W+\Delta)} - P_{V(W)}\right)\right\|_2 \\
&\leq \quad 2\max(\left\|P_{U(W+\Delta)} - P_{U(W)}\right\|, \left\|P_{V(W+\Delta)} - P_{V(W)}\right\|) \\
&\leq \quad \tfrac{8}{\sigma_r}||\Delta||_2,
\end{aligned}
$$

where we used the corollary given earlier. This proves part 1) of Proposition 2.

We now turn to part 2), where we wish to prove that

$$\left\|\mathcal{P}_{T(W)^\perp}(\Delta)\right\|_2 \leq \frac{4}{\sigma}||\Delta||_2^2.$$

This is a direct consequence of Proposition 3. Indeed, we have that

$$||\mathcal{P}_{T(W)^\perp}(\Delta)||_2 = ||(I - P_{U(W)}\Delta(I - P_{V(W)})||_2,$$

which – using the contra-positive of Proposition 3 – concludes the proof, since we assumed that $W + \Delta$ was a rank r matrix.

2.4.3 Curvature of the likelihood

Throughout the rest of the supplementary materials we will make use of the following notations (they are also in our summary of notations).

Let $w = \max(1, \frac{1}{\gamma})$ and set $D = \max(1, \frac{\nu\alpha}{3\beta(2-\nu)})$. We assume that

$$\gamma \in \left[\frac{3\xi(T)\beta(2-\nu)}{\nu\alpha}, \frac{\nu\alpha}{2\mu(\Omega)\beta(2-\nu)}\right],$$

so that $w \leq \frac{D}{\xi(T)}$.

In order to bound the error terms, it is necessary to study the likelihood gradient at the true parameters. In particular, we want to know how "close" (here taken in the $||\cdot||_2$ sense) to the true parameters the estimates have to be in order to achieve a particular error bound.

To that end we introduce the following matrix valued function:

$$\begin{array}{cccc} \mathcal{F}_{\Sigma_Z^n} : & \mathbb{R}^{(m+p)\times p} & \to & \mathbb{R}^{(m+p)\times p} \\ & M = \begin{pmatrix} M_X \\ M_{ZX} \end{pmatrix} & \mapsto & \begin{pmatrix} \mathcal{F}_{\Sigma_Z^n, X}(M) \\ \mathcal{F}_{\Sigma_Z^n, ZX}(M) \end{pmatrix} \triangleq \begin{pmatrix} M_X^{-1} + M_X^{-1} M_{ZX}^T \Sigma_Z^n M_{ZX} M_X^{-1} \\ -2\Sigma_Z^n M_{ZX} M_X^{-1} \end{pmatrix}. \end{array}$$

We can now express the following proposition which will be used later in order to bound the error terms.

Proposition 5. *Consider the Taylor expansion of $\mathcal{F}_{\Sigma_Z^n}$ at $K^* = \begin{pmatrix} K_X^* \\ K_{ZX}^* \end{pmatrix}$:*

$$\mathcal{F}_{\Sigma_Z^n}(K^* + \Delta) = \mathcal{F}_{\Sigma_Z^n} K^* + d(\mathcal{F}_{\Sigma_Z^n} K^*)\Delta + R_{K^*}(\Delta),$$

(note that in order to avoid cluttered notations we do not write $R_{K^*,\Sigma_Z^n}(\Delta)$, but the reader should not forget that there is a dependency here.). Write $\psi_Z \triangleq ||\Sigma_Z^n||_2$, $\psi_X^* \triangleq ||K_X^{*-1}||_2$ and $\phi_{ZX}^* \triangleq ||K_{ZX}^*||_2$. If $||\Delta||_2 \leq \frac{1}{2\psi_X^*}$ then

$$||R_{K^*}(\Delta)||_2 \leq 4\psi_X^* \psi^2 ||\Delta||_2^2,$$

where

$$\psi \triangleq 2\psi_X^* \sqrt{\left(1 + 6\frac{\psi_Z}{\psi_X^*}\left(1 + \psi_X^* \phi_{ZX}^*\right)^2\right)}.$$

Proof. We start by recalling that for any two matrices A, B (of compatible dimensions) we have the following inequalities:

$$\max(||A||_2, ||B||_2) \leq ||C||_2 \leq ||A||_2 + ||B||_2,$$

where $C = \begin{pmatrix} A \\ B \end{pmatrix}$.

We use arguments that are similar to Wytock & Kolter (2013), Lemma 1. By the mean value theorem, there exists a real t, $0 \leq t \leq 1$, such that $R_{K^*}(\Delta) = d^2(\mathcal{F}_{\Sigma_Z^n}(K^* + t\Delta); \Delta)$, which is the second derivative at $K^* + t\Delta$ evaluated at Δ. Given the inequality above, it is clear that, in order to obtain a bound on the norm of the remainder, it is enough to bound the sum $||R_{X,K^*}(\Delta_X)||_2 + ||R_{ZX,K^*}(\Delta_{ZX})||_2$. This is achieved by bounding both terms individually.

A tedious calculation (similar in all points to the one given in the appendix of Wytock & Kolter (2013)) yields the following expressions:

$$\begin{aligned}
d^2(\mathcal{F}_{X,\Sigma_Z^n}(M;\Delta)) \quad &= \quad 2(M_X^{-1}\Delta_X M_X^{-1}\Delta_X M_X^{-1} + M_X^{-1}\Delta_X M_X^{-1}\Delta_X M_X^{-1} M_{ZX}^T \Sigma_Z^n M_{ZX} M_X^{-1} + \\
&\quad M_X^{-1}\Delta_X M_X^{-1} M_{ZX}^T \Sigma_Z^n M_{ZX} M_X^{-1}\Delta_X M_X^{-1} + \\
&\quad M_X^{-1} M_{ZX}^T \Sigma_Z^n M_{ZX} M_X^{-1}\Delta_X M_X^{-1}\Delta_X M_X^{-1} - \\
&\quad M_X^{-1}\Delta_X M_X^{-1}\Delta_{ZX}^T \Sigma_Z^n M_{ZX} M_X^{-1} - M_X^{-1}\Delta_X M_X^{-1} M_{ZX}^T \Sigma_Z^n \Delta_{ZX} M_X^{-1} - \\
&\quad M_X^{-1}\Delta_{ZX}^T \Sigma_Z^n M_{ZX} M_X^{-1}\Delta_X M_X^{-1} - M_X^{-1} M_{ZX}^T \Sigma_Z^n \Delta_{ZX} M_X^{-1}\Delta_X M_X^{-1} + \\
&\quad M_X^{-1}\Delta_{ZX}^T \Sigma_Z^n \Delta_{ZX} M_X^{-1}). \\
d^2(\mathcal{F}_{ZX,\Sigma_Z^n}(M;\Delta)) \quad &= \quad 2(-2\Sigma_Z^n M_{ZX} M_X^{-1}\Delta_X M_X^{-1}\Delta_X M_X^{-1} + 2\Sigma_Z^n \Delta_{ZX} M_X^{-1}\Delta_X M_X^{-1})
\end{aligned}$$

We are interested in bounding $||d^2(\mathcal{F}_{\Sigma_Z^n}(K^*+t\Delta;\Delta))||_2$ and the terms $||(K_X^*+t\Delta_X)^{-1}||_2$, $||K_{ZX}^* + t\Delta_{ZX}||_2$ appear many times in these expressions. We use the assumption on $||\Delta||_2$ to show that $R_{K^*,\Sigma_Z^n}(\Delta)$ converges and to bound these two terms.

- Rewrite $(K_X^*+t\Delta_X)^{-1}$ as $K_X^{*-1}(I+tK_X^{*-1}\Delta_X)^{-1}$. Using the submultiplicative property of the spectral norm and the fact that $(I + tK_X^{*-1}\Delta_X)^{-1} = \sum_{i=0}^{\infty}(-1)^i(tK_X^{*-1}\Delta_X)^i$, we obtain :

$$||(K_X^* + t\Delta_X)^{-1}||_2 \leq \psi_X^* \frac{1}{1 - \psi_X^*||\Delta_X||_2};$$

which, by our assumptions on $||\Delta||_2$, implies

$$||(K_X^* + t\Delta_X)^{-1}||_2 \leq 2\psi_X^*.$$

- On the other hand, we have

$$||K_{ZX}^* + t\Delta_{ZX}||_2 \leq ||K_{ZX}^*||_2 + ||\Delta_{ZX}||_2 \leq \phi_{ZX}^* + \frac{1}{2\psi_X^*}.$$

Using these two inequalities, it is now straightforward to bound the remainder by bounding its summands independently. Putting together similar terms and rewriting the expression, we have

$$||R_{K^*}(\Delta)||_2 \leq 2(2\psi_X^*)^3 \left(1 + 6\frac{\psi_Z}{\psi_X^*}\left(1 + \psi_X^*\phi_{ZX}^*\right)^2\right) \max(||\Delta_X||_2^2, ||\Delta_{ZX}||_2^2),$$

which completes the proof. □

As a corollary, we can prove a result which is similar to Proposition 3.1 of Chandrasekaran et al. (2012), (suppl. mat.).

Corollary 2. *Suppose that γ is in the range required for identifiability. Let $g_\gamma(\Delta_S, \Delta_L) \leq \frac{1}{2\psi_X^*}\frac{1}{1+\frac{\alpha}{6\beta}}$, for any (Δ_S, Δ_L) with $\Delta_S \in \Omega$. Then we have*

$$g_\gamma(\mathcal{A}^\dagger R_{K^*}\mathcal{A}(\Delta_S, \Delta_L)) \leq 4\frac{D}{\xi(T)}\psi_X^*\psi^2(1 + \frac{\alpha}{6\beta})^2 g_\gamma(\Delta_S, \Delta_L)^2.$$

Proof. Following the proof given in Chandrasekaran et al. (2012), we derive a result which relates the dual norm g_γ to the spectral norm :

$$\begin{aligned} ||\mathcal{A}(\Delta_S, \Delta_L)||_2 &\leq ||\Delta_S||_2 + ||\Delta_L||_2 \\ &\leq \gamma\mu(\Omega)\tfrac{||\Delta_S||_\infty}{\gamma} + ||\Delta_L||_2 \\ &\leq (1 + \gamma\mu(\Omega))g_\gamma(\Delta_S, \Delta_L) \\ &\leq (1 + \tfrac{\alpha}{6\beta})g_\gamma(\Delta_S, \Delta_L) \\ &\leq \tfrac{1}{2\psi_X^*}, \end{aligned}$$

where we used the range of γ and the assumption on g_γ. Therefore, the assumptions of Proposition 5 are met and we have:

$$||R_{K^*}(\mathcal{A}(\Delta_S, \Delta_L)||_2 \leq 4\psi_X^* \psi^2 ||\mathcal{A}(\Delta_S, \Delta_L)||_2^2$$

which implies

$$||R_{K^*}(\mathcal{A}(\Delta_S, \Delta_L)||_2 \leq 4\psi_X^* \psi^2 (1 + \frac{\alpha}{6\beta})^2 g_\gamma(\Delta_S, \Delta_L)^2.$$

Finally, from the definition of $\mathcal{A}^\dagger$ of g_γ, we have

$$g_\gamma(\mathcal{A}^\dagger R_{K^*}(\mathcal{A}(\Delta_S, \Delta_L)) = \max(\frac{1}{\gamma}||R_{K^*}(\mathcal{A}(\Delta_S, \Delta_L)||_\infty, ||R_{K^*}(\mathcal{A}(\Delta_S, \Delta_L)||_2).$$

Recall also the definition of $w = \max(1/\gamma, 1)$ and the fact that $w \leq \frac{D}{\xi(T)}$. Then,

$$\max(\frac{1}{\gamma}||R_{K^*}(\mathcal{A}(\Delta_S, \Delta_L)||_\infty, ||R_{K^*}(\mathcal{A}(\Delta_S, \Delta_L)||_2 \leq \frac{D}{\xi(T)}||R_{K^*}(\mathcal{A}(\Delta_S, \Delta_L)||_2,$$

and the result follows from the bound on $||R_{K^*}(\mathcal{A}(\Delta_S, \Delta_L)||_2$. □

2.5 Consistency

This section studies a variant of our estimator in which the constraint $L_X \succeq 0$ is removed:

$$(\hat{S}_X, \hat{L}_X, \hat{S}_{ZX}, \hat{L}_{ZX}) = \\ \arg\min_{S_X, L_X, S_{ZX}, L_{ZX}} \ell(S_{ZX} - L_{ZX}, S_X - L_X; \Sigma_O^n) + \lambda(\gamma \|S\|_1 + \|L\|_*) \\ \text{s.t } S_X - L_X \succ 0 \text{ and } S = \begin{pmatrix} S_X \\ S_{ZX} \end{pmatrix}, L = \begin{pmatrix} L_X \\ L_{ZX} \end{pmatrix}. \quad (2.2)$$

Our proof of consistency follows the same pattern as the one in Chandrasekaran et al. (2012). Because the likelihood is different and because we consider rectangular matrices, the actual statements of the theorems and propositions are almost always different. In some

cases, however, the proof is easily adapted from Chandrasekaran et al. (2012) without any major difficulty. For that reason, we will focus on points that are critical to this particular analysis.

Compared to the standard approach to prove the consistency of lasso-type estimators, such as the one used in Ravikumar et al. (2011), Wytock & Kolter (2013), this proof strategy is more involved. Indeed, since the variety of sparse matrices has zero-curvature in its smooth points, tangent space constraints and variety constraints are equivalent. Unfortunately, we also have to deal with the rank variety which has non-zero curvature, hence the need to control for all the tangent spaces that are close to the nominal T.

A detailed description of the proof strategy, along with the rationale behind this approach, is given in Chandrasekaran et al. (2012). The key steps are the following:

- We start by considering a version of (2.2) in which the tangent space constraints are enforced explicitly. In its resort to Brouwer's fixed point theorem, this part is fairly similar to the proof given in Ravikumar et al. (2011), Wytock & Kolter (2013).

- We then consider a problem in which the variety constraints are explicitly enforced and show that the optimum of that non-convex problem is algebraically consistent.

- We then show that the optima of the variety constrained and tangent space constrained problems are identical.

- Finally, we derive conditions for the optimum of (2.2) to be identical to the optimum of the tangent space constrained problem.

2.5.1 Statement of the result

First, let us establish the result we seek to prove.

We define the following quantities : $D = \max(1, \frac{\nu\alpha}{3\beta(2-\nu)})$, $\psi_Z = \|\Sigma_Z^n\|_2$, $\psi_X^* = \left\|K_X^{*-1}\right\|_2$ and $\phi_{ZX}^* = \|K_{ZX}^*\|_2$, $\psi = 2\psi_X^*\sqrt{\left(1 + 6\frac{\psi_Z}{\psi_X^*}\left(1 + \psi_X^*\phi_{ZX}^*\right)^2\right)}$. We also set

$$C_1 = \frac{48}{\alpha} + \frac{1}{\psi_X^{*2}\left(1 + 2\frac{\psi_Z}{\psi_X^*}\left(1 + \psi_X^*\phi_{ZX}^*\right)^2\right)},$$

$$C_2 = \left(1 + \frac{24(2-\nu)}{\nu}\right) C_1^2 D\psi_X^{*2}\left(1 + 2\frac{\psi_Z}{\psi_X^*}\left(1 + \psi_X^*\phi_{ZX}^*\right)^2\right),$$

$$C_3 = C_1 + \frac{3\alpha C_1^2(2-\nu)}{4(3-\nu)}, C_4 = \max\{C_2, C_3\}, C_5 = \frac{C_1\nu\alpha}{\beta(2-\nu)},$$

$$C_6 = \frac{\alpha\nu}{32(3-\nu)D}\frac{1}{1+\frac{\alpha}{6\beta}}\min\left(\frac{1}{4\psi_X^*}, \frac{\alpha\xi(T)}{512D\psi_X^*\psi^2(1+\frac{\alpha}{6\beta})}\right).$$

Finally, set:

$$\delta_n = \sqrt{\frac{256\psi_X^{*2}pM}{n}},$$

with $M = \max\left(1, \frac{\psi_Z}{4\psi_X^*}(1 + \sqrt{\frac{m}{p}})^2\right)$. And, let

$$\lambda_n = \frac{6D(2-\nu)\delta_n}{\xi(T)\nu}.$$

We want to show the following.

Theorem 1. *(Algebraic Consistency)*
Suppose that Assumptions 1 and 2 hold and that we are given n i.i.d. samples drawn according to (1.3)*. Further assume that the following hold:*

1. $n \geq \frac{pM}{\xi(T)^4}\max\left(2, \frac{256\psi_X^{*2}}{C_6^2}\right)$.
2. *Let the minimum non-zero singular value of L^* be such that*

$$\sigma \geq \frac{C_4\lambda_n}{\xi(T)^2}.$$

3. Let the minimum magnitude nonzero entry θ of S^* be such that

$$\theta \geq \frac{C_5 \lambda_n}{\mu(\Omega)}.$$

Then, with probability greater than $1 - \max\left(2\exp\left(-pM\right), \exp\left(-\frac{4\psi_X^*}{\psi_Z} pM\right)\right)$, *we have that*

1. $sign(\hat{S}) = sign(S^*)$ *and* $rank(\hat{L}) = rank(L^*)$

2.

$$\max\left(\frac{1}{\gamma}\left\|\hat{S} - S^*\right\|_\infty, \left\|\hat{L} - L^*\right\|_2\right) \leq \frac{32(3-\nu)}{3\alpha(2-\nu)}\lambda_n.$$

In particular, due to the form taken by δ_n,

$$\max\left(\frac{1}{\gamma}\left\|\hat{S} - S^*\right\|_\infty, \left\|\hat{L} - L^*\right\|_2\right) \leq C\frac{1}{\xi(T)}\sqrt{\frac{pM}{n}},$$

with $C = \frac{1024(3-\nu)D\psi_X^*}{\nu\alpha}$.

2.5.2 Tangent space constraints

We consider

$$\begin{aligned}(\hat{S}_\Omega, \hat{L}_{T'}) \quad = \quad & \arg\min_{S,L} -\log\det(S_X - L_X) + tr\left(\Sigma_X^n(S_X - L_X)\right) + 2tr\left(\Sigma_{ZX}^n(S_{ZX} + L_{ZX})^T\right) \\ & + tr(\Sigma_Z^n(S_{ZX} + L_{ZX})(S_X - L_X)^{-1}(S_{ZX} + L_{ZX})^T) \\ & \text{s.t. } S_X - L_X \succ 0,\ S \in \Omega,\ L \in T' \\ & \text{where } S = \begin{pmatrix} S_X \\ S_{ZX} \end{pmatrix}, L = \begin{pmatrix} L_X \\ L_{ZX} \end{pmatrix}; \end{aligned} \tag{2.3}$$

for some T'. The goal of this section is to show that if T' is sufficiently close to the nominal T (as measured by $\rho(T, T')$), then the error terms are bounded.

Let $C_{T'} = \mathcal{P}_{T'}(L^*)$ be the orthogonal projection of the nominal low-rank matrix onto the linear subspace T'.

Proposition 6. *Let the errors* (Δ_S, Δ_L) *be defined as above and assume that* T' *is such that* $\rho(T, T') \leq \frac{\xi(T)}{2}$. *Define*

$$E_n = \begin{pmatrix} \Sigma_X^n \\ 2\Sigma_{ZX}^n \end{pmatrix} - \mathcal{F}_{\Sigma_Z^n}(\begin{pmatrix} K_X^* \\ K_{ZX}^* \end{pmatrix})$$

and

$$r = \max\left(\frac{8}{\alpha}\left(g_\gamma(\mathcal{A}^\dagger E_n) + g_\gamma(\mathcal{A}^\dagger \mathcal{I}^*_{\Sigma_Z^n} \mathcal{C}_{T'}) + \lambda_n\right), ||\mathcal{C}_{T'}||_2\right).$$

If

$$r \leq \min\left(\frac{1}{4\psi_X^*}\frac{1}{1+\frac{\alpha}{6\beta}}, \frac{\alpha\xi(T)}{128D\psi_X^*\psi^2(1+\frac{\alpha}{6\beta})^2}\right),$$

then

$$g_\gamma(\Delta_S, \Delta_L) \leq 2r.$$

Proof. The proof is very similar to the one given in Chandrasekaran et al. (2012) (suppl. mat. Prop. 3.2) but differs in a few different places. In order to spare the reader the trouble of going back and forth between Chandrasekaran et al. (2012) and this proof, we give all the details here, even for those parts that are identical.

Start by noticing that the objective function (2.3) is strictly convex: we showed earlier that the log-likelihood is convex; the tangent constraints ensure that it is *strictly* convex. As such, it has a unique minimum. By applying the optimality conditions of this function at $(\hat{S}_\Omega, \hat{L}_{T'})$, there exists two Lagrange multipliers $Q_{\Omega^\perp} \in \Omega^\perp$, $Q_{T'^\perp} \in T'^\perp$ such that

$$\begin{pmatrix} \Sigma_X^n \\ 2\Sigma_{ZX}^n \end{pmatrix} - \mathcal{F}_{\Sigma_Z^n}(\hat{S}_\Omega - \hat{L}_{T'}) + Q_{\Omega^\perp} \in \lambda_n \gamma \partial ||\hat{S}_\Omega||_1,$$

and

$$\begin{pmatrix} \Sigma_X^n \\ 2\Sigma_{ZX}^n \end{pmatrix} - \mathcal{F}_{\Sigma_Z^n}(\hat{S}_\Omega - \hat{L}_{T'}) + Q_{T'^\perp} \in \lambda_n \partial ||\hat{L}_{T'}||_*.$$

In particular, one checks that

$$\mathcal{P}_{\Omega}\left[\begin{pmatrix} \Sigma_X^n \\ 2\Sigma_{ZX}^n \end{pmatrix} - \mathcal{F}_{\Sigma_Z^n}(\hat{S}_{\Omega} - \hat{L}_{T'})\right] = Z_{\Omega},$$

and

$$\mathcal{P}_{T'}\left[\begin{pmatrix} \Sigma_X^n \\ 2\Sigma_{ZX}^n \end{pmatrix} - \mathcal{F}_{\Sigma_Z^n}(\hat{S}_{\Omega} - \hat{L}_{T'})\right] = Z_{T'},$$

with $Z_{\Omega} \in \Omega$, $Z_{T'} \in T'$ and $||Z_{\Omega}||_{\infty} \leq \lambda_n \gamma, ||Z_{T'}||_2 \leq \lambda_n$. Write $Z = (Z_{\Omega}, Z_{T'})$. We then have

$$\mathcal{P}_{\mathcal{Y}}\mathcal{A}^{\dagger}\left[\begin{pmatrix} \Sigma_X^n \\ 2\Sigma_{ZX}^n \end{pmatrix} - \mathcal{F}_{\Sigma_Z^n}(\hat{S}_{\Omega} - \hat{L}_{T'})\right] = Z,$$

with $g_{\gamma}(Z) \leq 2\lambda_n$. Moreover, because the optimum is unique, $(\hat{S}_{\Omega}, \hat{L}_{T'})$ is the unique solution to this equation.

We now rewrite the difference

$$\begin{pmatrix} \Sigma_X^n \\ 2\Sigma_{ZX}^n \end{pmatrix} - \mathcal{F}_{\Sigma_Z^n}(\hat{S}_{\Omega} - \hat{L}_{T'})$$

in terms of the errors (Δ_S, Δ_L).

To that end remark that, by definition, $K^* = S^* - L^*$, so that

$$\begin{aligned}
\begin{pmatrix} \Sigma_X^n \\ 2\Sigma_{ZX}^n \end{pmatrix} - \mathcal{F}_{\Sigma_Z^n}(\hat{S}_{\Omega} - \hat{L}_{T'}) &= \begin{pmatrix} \Sigma_X^n \\ 2\Sigma_{ZX}^n \end{pmatrix} - \mathcal{F}_{\Sigma_Z^n}(K^* + \mathcal{A}(\Delta_S, \Delta_L)) \\
&= \begin{pmatrix} \Sigma_X^n \\ 2\Sigma_{ZX}^n \end{pmatrix} - \mathcal{F}_{\Sigma_Z^n}K^* - R_{K^*}(\mathcal{A}(\Delta_S, \Delta_L)) + \mathcal{I}^*_{\Sigma_Z^n}\mathcal{A}(\Delta_S, \Delta_L) \\
&\triangleq E_n - R_{K^*}(\mathcal{A}(\Delta_S, \Delta_L)) + \mathcal{I}^*_{\Sigma_Z^n}\mathcal{A}(\Delta_S, \Delta_L) \\
&= E_n - R_{K^*}(\mathcal{A}(\Delta_S, \Delta_L)) + \mathcal{I}^*_{\Sigma_Z^n}\mathcal{A}\mathcal{P}_{\mathcal{Y}}(\Delta_S, \Delta_L) + \mathcal{I}^*_{\Sigma_Z^n}\mathcal{C}_{T'}.
\end{aligned}$$

Now, since T' is such that $\rho(T, T') \leq \frac{\xi(T)}{2}$, we have from Proposition 1 that the operator $\mathcal{B} = (\mathcal{P}_{\mathcal{Y}} \mathcal{A}^{\dagger} \mathcal{I}^{*}_{\Sigma^n_Z} \mathcal{A} \mathcal{P}_{\mathcal{Y}})^{-1}$ is an homeomorphism from $\mathcal{Y} \to \mathcal{Y}$.

Following the construction of Chandrasekaran et al. (2012), we consider the following function

$$\begin{aligned} F: \quad & \mathcal{Y} && \to \mathcal{Y} \\ & (\delta_S, \delta_L) && \mapsto (\delta_S, \delta_L) - \mathcal{B}\left(\mathcal{P}_{\mathcal{Y}} \mathcal{A}^{\dagger} \left[E_n - R_{K^*}(\mathcal{A}(\delta_S, \delta_L)) + \mathcal{I}^{*}_{\Sigma^n_Z} \mathcal{A} \mathcal{P}_{\mathcal{Y}}(\delta_S, \delta_L) + \mathcal{I}^{*}_{\Sigma^n_Z} \mathcal{C}_{T'}\right] - Z\right). \end{aligned}$$

Now, assume for a minute that there exist a fixed point (δ_S, δ_L) of F. By construction of F, (δ_S, δ_L) is a fixed point if and only if

$$0 = \mathcal{B}\left(\mathcal{P}_{\mathcal{Y}} \mathcal{A}^{\dagger} \left[E_n - R_{K^*}(\mathcal{A}(\delta_S, \delta_L)) + \mathcal{I}^{*}_{\Sigma^n_Z} \mathcal{A} \mathcal{P}_{\mathcal{Y}}(\delta_S, \delta_L) + \mathcal{I}^{*}_{\Sigma^n_Z} \mathcal{C}_{T'}\right] - Z\right).$$

Since $\mathcal{B}$ is bijective, we conclude that (δ_S, δ_L) is a fixed-point if and only if

$$\mathcal{P}_{\mathcal{Y}} \mathcal{A}^{\dagger} \left[E_n - R_{K^*}(\mathcal{A}(\delta_S, \delta_L)) + \mathcal{I}^{*}_{\Sigma^n_Z} \mathcal{A} \mathcal{P}_{\mathcal{Y}}(\delta_S, \delta_L) + \mathcal{I}^{*}_{\Sigma^n_Z} \mathcal{C}_{T'}\right] = Z.$$

Going back to the definition of Z given above, we see that, by construction, the fixed-point must be $\mathcal{P}_{\mathcal{Y}}(\Delta_S, \Delta_L)$. Moreover, recall that the solution to that equation is unique.

We now focus on showing that such a fixed-point exists. In particular, we show that this unique fixed-point lies in

$$\mathbb{B}_r = \{(\delta_S, \delta_L) | g_{\gamma}(\delta_S, \delta_L) \leq r, (\delta_S, \delta_L) \in \mathcal{Y}\}.$$

To that end, let us show that $\mathcal{B}$ maps this closed ball onto itself and conclude the existence of a fixed-point by Brouwer's theorem.

First of all, F simplifies to

$$\mathcal{B}\left(\mathcal{P}_{\mathcal{Y}} \mathcal{A}^{\dagger} \left[-E_n + R_{K^*}(\mathcal{A}(\delta_S, \delta_L)) - \mathcal{I}^{*}_{\Sigma^n_Z} \mathcal{C}_{T'}\right] + Z\right)$$

Now, we can apply Proposition 1 once more:

$$\begin{aligned} g_\gamma(\delta_S, \delta_L) &\le \frac{2}{\alpha} g_\gamma \left(\mathcal{P}_{\mathcal{Y}} \mathcal{A}^\dagger \left[E_n - R_{K^*}(\mathcal{A}(\delta_S, \delta_L)) + \mathcal{I}^*_{\Sigma^n_Z} \mathcal{C}_{T'} \right] - Z \right) \\ &\le \frac{4}{\alpha} \left(g_\gamma \left(\mathcal{A}^\dagger \left[E_n - R_{K^*}(\mathcal{A}(\delta_S, \delta_L)) + \mathcal{I}^*_{\Sigma^n_Z} \mathcal{C}_{T'} \right] \right) + \lambda_n \right) \\ &\le \frac{r}{2} + \frac{4}{\alpha} g_\gamma \left(\mathcal{A}^\dagger R_{K^*}(\mathcal{A}(\delta_S, \delta_L + \mathcal{C}_{T'})) \right). \end{aligned}$$

The second inequality uses the fact that $g_\gamma(\mathcal{P}_{\mathcal{Y}}(\cdot, \cdot)) \le 2 g_\gamma(\cdot, \cdot)$, while the last one uses the definition of r.

Let us now bound the second summand of the last inequality, $\frac{4}{\alpha} g_\gamma \left(\mathcal{A}^\dagger R_{K^*}(\mathcal{A}(\delta_S, \delta_L + \mathcal{C}_{T'})) \right)$, by $\frac{r}{2}$. By our assumptions on r, we can apply Proposition 2:

$$\begin{aligned} \frac{4}{\alpha} g_\gamma \left(\mathcal{A}^\dagger R_{K^*}(\mathcal{A}(\delta_S, \delta_L + \mathcal{C}_{T'})) \right) &\le \frac{16 D \psi^*_X \psi^2 (1 + \frac{\alpha}{6\beta})^2 (g_\gamma(\delta_S, \delta_L) + ||\mathcal{C}_{T'}||_2)^2}{\alpha \xi(T)} \\ &\le \frac{64 D \psi^*_X \psi^2 (1 + \frac{\alpha}{6\beta})^2 r^2}{\alpha \xi(T)} \\ &\le \frac{64 D \psi^*_X \psi^2 (1 + \frac{\alpha}{6\beta})^2 r}{\alpha \xi(T)} \frac{\alpha \xi(T)}{128 D \psi^*_X \psi^2 (1 + \frac{\alpha}{6\beta})^2} \\ &\le \frac{r}{2}. \end{aligned}$$

To go from the first line to the second, we used the definition of r and the fact that $g_\gamma(\delta_S, \delta_L)$ lies in the ball $\mathbb{B}_r$. Going from line 2 to 3 uses the assumption on r. Combining this result with the previous sequence of inequalities, we have shown that $F(\delta_S, \delta_L) \le r$, for any (δ_S, δ_L) in $\mathbb{B}_r$. It follows that F maps the closed ball $\mathbb{B}_r$ onto itself and we are now within the range of applicability of Brouwer's fixed-point theorem. Subsequently, F admits a fixed-point within $\mathbb{B}_r$ and, as argued earlier, this fixed-point must be $\mathcal{P}_{\mathcal{Y}}(\Delta_S, \Delta_L)$. Therefore, $g_\gamma(\mathcal{P}_{\mathcal{Y}}(\Delta_S, \Delta_L)) \le r$.

Finally, we have

$$\begin{aligned} g_\gamma(\Delta_S, \Delta_L) &\leq g_\gamma(\mathcal{P}_{\mathcal{Y}}(\Delta_S, \Delta_L)) + ||\mathcal{C}_{T'}||_2 \\ &\leq 2r. \end{aligned}$$

□

2.5.3 Variety constraints

We now turn to a variant of the problem in which both the rank and the sparsity pattern are enforced explicitly. This amounts to minimising the objective function within the following non-convex constraint set:

$$\begin{aligned} \mathcal{M} = \{(S, L) | S \in \Omega(S^*), \text{rank}(L) \leq \text{rank}(L^*), \\ \|\mathcal{P}_{T^\perp}(L - L^*)\|_2 \leq \frac{\xi(T)\lambda_n}{D\psi_X^{*2}\left(1 + 2\frac{\psi_Z}{\psi_X^*}\left(1 + \psi_X^*\phi_{ZX}^*\right)^2\right)}, \\ g_\gamma(\mathcal{A}^\dagger \mathcal{I}^*_{\Sigma_Z^n} \mathcal{A}(S - S^*, L^* - L)) \leq 11\lambda_n\}. \end{aligned}$$

We denote by $(\hat{S}_{\mathcal{M}}, \hat{L}_{\mathcal{M}})$ the solution to our problem under these constraints, *i.e.*:

$$\begin{aligned} (\hat{S}_{\mathcal{M}}, \hat{L}_{\mathcal{M}}) \;\; = \;\; & \arg\min_{S,L} -\log\det(S_X - L_X) + tr\left(\Sigma_X^n(S_X - L_X)\right) + 2tr\left(\Sigma_{ZX}^n(S_{ZX} + L_{ZX})^T\right) \\ & + tr(\Sigma_Z^n(S_{ZX} + L_{ZX})(S_X - L_X)^{-1}(S_{ZX} + L_{ZX})^T) \\ & \text{s.t. } S_X - L_X \succ 0, \; (S, L) \in \mathcal{M} \\ & \text{where } S = \begin{pmatrix} S_X \\ S_{ZX} \end{pmatrix}, L = \begin{pmatrix} L_X \\ L_{ZX} \end{pmatrix}; \end{aligned} \tag{2.4}$$

We start by proving a preliminary result that bounds the spectral norm of the FIM evaluated at the nominal parameters. This simple bound will be used later in this section.

Proposition 7. *Recall that* $K^* = \begin{pmatrix} K_X^* \\ K_{ZX}^* \end{pmatrix}$ *and* $\mathcal{I}^*_{\Sigma_Z^n} \triangleq \mathcal{I}_{\Sigma_Z^n}(K^*)$.

$$||\mathcal{I}^*_{\Sigma_Z^n}||_2 \leq \psi_X^{*2}\left(1 + 2\frac{\psi_Z}{\psi_X^*}\left(1+\psi_X^*\phi_{ZX}^*\right)^2\right).$$

Proof. As shown earlier, we have

$$\mathcal{I}^*_{\Sigma_Z^n} = -\begin{pmatrix} K_X^{*-1}\otimes K_X^{*-1} & 0 \\ 0 & 0 \end{pmatrix} - 2K_X^{*-1}\otimes\begin{pmatrix} K_X^{*-1}K_{ZX}^{*T}\Sigma_Z^n K_{ZX}^* K_X^{*-1} & -K_X^{*-1}K_{ZX}^{*T}\Sigma_Z^n \\ \cdot & \Sigma_Z^n \end{pmatrix}.$$

Therefore,

$$\begin{aligned} ||\mathcal{I}^*_{\Sigma_Z^n}||_2 &\leq {\psi_X^*}^2 + 2\psi_X^*\left\|\begin{pmatrix} K_X^{*-1}K_{ZX}^{*T}\Sigma_Z^n K_{ZX}^* K_X^{*-1} & -K_X^{*-1}K_{ZX}^{*T}\Sigma_Z^n \\ \cdot & \Sigma_Z^n \end{pmatrix}\right\|_2 \\ &= \psi_X^{*2} + 2\psi_X^*\left\|\begin{pmatrix} K_X^{*-1}K_{ZX}^{*T}\sqrt{\Sigma_Z^n} \\ \sqrt{\Sigma_Z^n} \end{pmatrix}\begin{pmatrix} K_X^{*-1}K_{ZX}^{*T}\sqrt{\Sigma_Z^n} \\ \sqrt{\Sigma_Z^n} \end{pmatrix}^T\right\|_2 \\ &\leq \psi_X^{*2} + 2\psi_X^*(\psi_X^*\phi_{ZX}^*\sqrt{\psi_Z} + \sqrt{\psi_Z})^2 \\ &\leq \psi_X^{*2}(1 + 2\tfrac{\psi_Z}{\psi_X^*}(1+\psi_X^*\phi_{ZX}^*)^2). \end{aligned}$$

□

We now have the following results. Remark that because we derived different bounds on the curvature of the rank variety and on the spectral norm of $\mathcal{I}^*_{\Sigma_Z^n}$, the assumptions of these propositions differ from the one made in Chandrasekaran et al. (2012). However, given the above bound on $||\mathcal{I}^*_{\Sigma_Z^n}||_2$, the proof is a straightforward adaptation of the proof in Chandrasekaran et al. (2012).

Proposition 8. *(see Chandrasekaran et al. (2012) (suppl. mat.), Proposition 3.3) Consider any* $(S, L) \in \mathcal{M}$ *and let* $(\Delta_S, \Delta_L) = (S - S^*, L^* - L)$*. For* γ *in the range required*

for consistency and letting $C_1 = \frac{48}{\alpha} + \frac{1}{\psi_X^{*2}\left(1+2\frac{\psi_Z}{\psi_X^*}\left(1+\psi_X^*\phi_{ZX}^*\right)^2\right)}$, *we have that* $g_\gamma(\Delta_S, \Delta_L) \leq C_1\lambda_n$.

The following corollary shows that, under some suitable conditions, any solution to (2.4) is algebraically consistent. As a matter of fact, it holds for any pair in $\mathcal{M}$, irrespective of whether it is a solution to (2.4).

Here again, the proof of that corollary follows straightforwardly from the one given in Chandrasekaran et al. (2012). However, unlike the previous proposition, the statement of the following corollary does not obviously follow from Chandrasekaran et al. (2012) (suppl. mat.), Corollary 3.4. For that reason, we give details.

Corollary 3. *(see Chandrasekaran et al. (2012) (suppl. mat.), Corollary 3.4)*
Consider any pair $(S, L) \in \mathcal{M}$ *and, as before, let* $\Delta_S = S - S^*$, $\Delta_L = L^* - L$. *For* C_1 *defined as in the previous proposition, further define the following constants:*

- $C_2 = \left(1 + \frac{24(2-\nu)}{\nu}\right) C_1^2 D\psi_X^{*2} \left(1 + 2\frac{\psi_Z}{\psi_X^*}\left(1 + \psi_X^*\phi_{ZX}^*\right)^2\right)$;
- $C_3 = C_1 + \frac{3\alpha C_1^2(2-\nu)}{4(3-\nu)}$;
- $\sigma =$ *smallest non-zero singular value of* L^*;
- $C_4 = \max\{C_2, C_3\}$;
- $C_5 = \frac{C_1\nu\alpha}{\beta(2-\nu)}$;
- $T' = T(L)$ *and* $\mathcal{C}_{T'} = \mathcal{P}_{T'^\perp}(L^*)$.

Assume that $\sigma \geq \frac{C_4\lambda_n}{\xi(T)^2}$, *and suppose that the smallest entry in magnitude of* S^* *is greater than* $\frac{C_5\lambda_n}{\mu(\Omega)}$. *Further assume that* γ *in the acceptable range defined earlier. Then we have:*

1. L *has rank equal to* L^*, *and* $L_X \succeq 0$;

2. $sign(S) = sign(S^*)$;

3. $\|\mathcal{P}_{T^\perp}(\Delta_L)\|_2 \leq \frac{4\xi(T)\lambda_n}{73D\psi_X^{*2}\left(1+2\frac{\psi_Z}{\psi_X^*}\left(1+\psi_X^*\phi_{ZX}^*\right)^2\right)}$.

4. $\rho(T, T') \leq \frac{8\xi(T)}{73}$.

5. $g_\gamma(\mathcal{A}^\dagger\mathcal{I}_{\Sigma_Z}^*\mathcal{C}_{T'}) \leq \frac{\lambda_n \nu}{6(2-\nu)}$.

6. $\|\mathcal{C}_{T'}\|_2 \leq \frac{16(3-\nu)\lambda_n}{3\alpha(2-\nu)}$.

Proof. First of all, remark that

$$
\begin{aligned}
C_1 &\geq \frac{1}{\psi_X^{*2}\left(1+2\frac{\psi_Z}{\psi_X^*}\left(1+\psi_X^*\phi_{ZX}^*\right)^2\right)} \\
&\geq \frac{1}{w}\frac{1}{\psi_X^{*2}\left(1+2\frac{\psi_Z}{\psi_X^*}\left(1+\psi_X^*\phi_{ZX}^*\right)^2\right)} \\
&\geq \frac{\xi(T)}{D}\frac{1}{\psi_X^{*2}\left(1+2\frac{\psi_Z}{\psi_X^*}\left(1+\psi_X^*\phi_{ZX}^*\right)^2\right)}.
\end{aligned}
$$

We also have $\xi(T) \leq 1$ and, by Proposition 3, $||\Delta_L||_2 \leq C_1\lambda_n$. One can also check that, for $0 < \nu \leq \frac{1}{2}$, $\frac{24(2-\nu)}{\nu} \geq 72$.

By our assumptions,

$$\begin{aligned}\sigma &\geq \frac{C_4\lambda_n}{\xi(T)^2} \\ &\geq \frac{C_2\lambda_n}{\xi(T)^2} \\ &\geq \frac{73C_1^2 D\psi_X^{*2}\left(1+2\frac{\psi_Z}{\psi_X^*}\left(1+\psi_X^*\phi_{ZX}^*\right)^2\right)\lambda_n}{\xi(T)^2} \\ &\geq \frac{73C_1\lambda_n}{\xi(T)} \\ &\geq 8C_1\lambda_n \\ &\geq 8||\Delta_L||_2.\end{aligned}$$

So, the smallest singular value of L^* is greater than $4||L - L^*||_2$. Consequently, L and L^* have the same rank. Moreover, since $||L^*||_2 \geq \max(||L_X^*||_2, ||L_{ZX}^*||_2)$, $\sigma \leq$ (smallest singular value of L_X^*). Let us call σ_X the smallest eigenvalue of L_X^*. On the other hand, $||\Delta_L|| \geq \max(||\Delta_{L_X}||_2, ||\Delta_{L_{ZX}}||_2)$, so that

$$\sigma_X \geq \sigma \geq 8||\Delta_L||_2 \geq 8||\Delta_{L_X}||_2.$$

This implies that L_X and L_X^* have the same inertia, *i.e.* the same number of positive, null and negative eigenvalues.

This proves 1.

Now, since $\sigma > 4||\Delta_L||_2$, we can apply Proposition 2 and therefore

$$\begin{aligned}
||\mathcal{P}_{T^\perp}(\Delta_L)||_2 &\leq \frac{4||\Delta_L||_2^2}{\sigma} \\
&\leq \frac{4C_1^2\xi(T)^2\lambda_n^2}{C_2\lambda_n} \\
&\leq \frac{4\xi(T)^2\lambda_n}{73D\psi_X^{*2}\left(1+2\frac{\psi_Z}{\psi_X^*}\left(1+\psi_X^*\phi_{ZX}^*\right)^2\right)} \\
&\leq \frac{4\xi(T)\lambda_n}{73D\psi_X^{*2}\left(1+2\frac{\psi_Z}{\psi_X^*}\left(1+\psi_X^*\phi_{ZX}^*\right)^2\right)}.
\end{aligned}$$

This proves 3.

Now, we apply the other part of Proposition 2, so that

$$\begin{aligned}
\rho(T,T') &\leq \frac{8||\Delta_L||_2}{\sigma} \\
&\leq \frac{8C_1\xi(T)^2}{C_2\lambda_n} \\
&\leq \frac{8\xi(T)^2}{73C_1D\psi_X^{*2}\left(1+2\frac{\psi_Z}{\psi_X^*}\left(1+\psi_X^*\phi_{ZX}^*\right)^2\right)} \\
&\leq \frac{8\xi(T)}{73}.
\end{aligned}$$

This proves 4.

Let σ' be the smallest non-zero singular value of L.

$$\begin{aligned}
\sigma' &\geq \frac{C_2\lambda_n}{\xi(T)^2} - C_1\lambda_n \\
&\geq C_1\lambda_n\left(\frac{73C_1D\psi_X^{*2}\left(1+2\frac{\psi_Z}{\psi_X^*}\left(1+\psi_X^*\phi_{ZX}^*\right)^2\right)}{\xi(T)^2} - 1\right) \\
&\geq 5||\Delta_L||_2.
\end{aligned}$$

Here again, let us apply Proposition 2 but this time with σ':

$$\begin{aligned}
||\mathcal{C}_{T'}||_2 &\leq \frac{4||\Delta_L||_2^2}{\sigma'} \\
&\leq \frac{4C_1^2\lambda_n^2}{\frac{C_2\lambda_n}{\xi(T)^2} - C_1\lambda_n} \\
&= \frac{4C_1^2\xi(T)^2\lambda_n}{\frac{24(2-\nu)}{\nu}C_1^2 D\psi_X^{*2}\left(1 + 2\frac{\psi_Z}{\psi_X^*}\left(1+\psi_X^*\phi_{ZX}^*\right)^2\right) + C_1^2 D\psi_X^{*2}\left(1 + 2\frac{\psi_Z}{\psi_X^*}\left(1+\psi_X^*\phi_{ZX}^*\right)^2\right) - C_1\xi(T)^2} \\
&\leq \frac{4C_1^2\xi(T)^2\lambda_n}{\frac{24(2-\nu)}{\nu}C_1^2 D\psi_X^{*2}\left(1 + 2\frac{\psi_Z}{\psi_X^*}\left(1+\psi_X^*\phi_{ZX}^*\right)^2\right)} \\
&\leq \frac{\nu\xi(T)\lambda_n}{6(2-\nu)D\psi_X^{*2}\left(1 + 2\frac{\psi_Z}{\psi_X^*}\left(1+\psi_X^*\phi_{ZX}^*\right)^2\right)},
\end{aligned}$$

so that

$$g_\gamma(\mathcal{A}^\dagger\mathcal{I}^*_{\Sigma_Z^n}\mathcal{C}_{T'}) \leq w||\mathcal{I}^*_{\Sigma_Z^n}||_2||\mathcal{C}_{T'}||_2 \leq \frac{\nu\lambda_n}{6(2-\nu)}.$$

This proves 5.

Proof for 6. and 2. still remain. We have that

$$\begin{aligned}
\sigma' &\geq \frac{C_3\lambda_n}{\xi(T)^2} - C_1\lambda_n \\
&\geq \frac{3\alpha C_1^2(2-\nu)}{4(3-\nu)}\lambda_n,
\end{aligned}$$

which can be used to prove the desired bound on $||\mathcal{C}_{T'}||_2$. As before, we have

$$\begin{aligned}
||\mathcal{C}_{T'}||_2 &\leq \frac{4C_1^2\lambda_n^2}{\sigma'} \\
&\leq \frac{4C_1^2\lambda_n^2}{\frac{3\alpha C_1^2(2-\nu)}{4(3-\nu)}\lambda_n} \\
&\leq \frac{16(3-\nu)\lambda_n}{3\alpha(2-\nu)}.
\end{aligned}$$

This proves 6.

Finally, in order to prove 2., recall that $||\Delta_S||_\infty \leq \gamma C_1 \lambda_n$. In order to show that $sign(S^*) = sign(S)$ we need to show that the magnitude of the smallest entry of S^* is greater $||\Delta_S||_\infty$. Since γ is in the acceptable range, we know that

$$\gamma \leq \frac{\nu\alpha}{2\beta\mu(\Omega)(2-\nu)} \leq \frac{\nu\alpha}{\beta\mu(\Omega)(2-\nu)}.$$

So that, by the assumption on the magnitude of the smallest entry of S^*, θ, we have

$$\theta \geq \frac{C_1 \nu\alpha\lambda_n}{\mu(\Omega)\beta(2-\nu)} \geq C_1 \lambda_n \gamma.$$

□

2.5.4 From variety to tangent space constraints

In the previous section, we showed that any solution to the non-convex problem (2.4) was algebraically consistent (among other properties). This problem was non-convex due to the constraint $rank(L) \leq rank(L^*)$. Here, we consider a different function where the constraint on the rank of L is replaced by another one $L \in T_{\mathcal{M}} \triangleq T(\hat{L}_{\mathcal{M}})$, where $(\hat{S}_{\mathcal{M}}, \hat{L}_{\mathcal{M}})$ is any solution to (2.4).

We denote by $(\hat{S}_{\mathcal{M}}, \hat{L}_{T_{\mathcal{M}}})$ the solution to that modified convex problem.

The following proposition shows that, under suitable conditions, the solution to that modified problem is the same as the variety constrained one. In particular, it is algebraically consistent.

The proof is lengthy and technical. However, we tailored the assumptions of the previous results (the definitions of C_2, C_3 in the previous corollary in particular) so as to achieve bounds that are identical – or tighter – than the ones derived in Chandrasekaran et al. (2012) (Prop. 3.5 of the suppl. materials). We therefore refer the reader to Chandrasekaran et al. (2012) for the proof. Given the result we proved in the previous section, understanding their proof does not raise any challenges.

Proposition 9. *(see Chandrasekaran et al. (2012) (suppl. mat.), Proposition 3.5)*
Let γ be in the correct range. Suppose that the minimum non-zero singular value of L^* is such $\sigma \geq \frac{C_4 \lambda_n}{\xi(T)^2}$, and suppose that the smallest entry in magnitude of S^* is greater than $\frac{C_5 \lambda_n}{\mu(\Omega)}$ (with $C_{4,5}$ defined as above). Let $g_\gamma(\mathcal{A}^\dagger E^n) \leq \frac{\lambda_n \nu}{6(2-\nu)}$. Further suppose that

$$\lambda_n \leq \frac{3\alpha(2-\nu)}{16(3-\nu)} \frac{1}{1+\frac{\alpha}{6\beta}} \min\left(\frac{1}{4\psi_X^*}, \frac{\alpha \xi(T)}{128 D \psi_X^* \psi^2 (1+\frac{\alpha}{6\beta})}\right).$$

Then we have $(\hat{S}_\Omega, \hat{L}_{T_\mathcal{M}}) = (\hat{S}_\mathcal{M}, \hat{L}_\mathcal{M})$.

Corollary 4. *Under the assumptions of Proposition 9, we have that rank$(\hat{L}_{T_\mathcal{M}})$ = rank(L^*) and that $T(\hat{L}_{T_\mathcal{M}}) = T_\mathcal{M}$. Similarly, sign$(\hat{S}_\Omega)$ = sign(S^*).*

2.5.5 From tangent space constraints to problem (2.2)

At this stage, we know under which conditions the problem with tangent space constraints is algebraically consistent. The following result derives a condition under which this constrained problem admits the same solution as (2.2). Recall that (2.2) differs from the original problem by the removal of the constraint $L_X \succeq 0$.

Lemma 1. *(see, Chandrasekaran et al. (2012) (suppl. mat.), Lemma 3.7)*
Let $(\hat{S}_\Omega, \hat{L}_{T_\mathcal{M}})$ be defined as in the previous section. Suppose that the assumptions of Proposition 9 hold. Further assume that

$$g_\gamma(\mathcal{A}^\dagger R_{K^*} \mathcal{A}(\Delta_S, \Delta_L)) \leq \frac{\lambda_n \nu}{6(2-\nu)}.$$

Then $(\hat{S}_\Omega, \hat{L}_{T_\mathcal{M}})$ is also the unique optimum to problem (2.2).

2.5.6 Bounding the error terms

We start by recalling a useful result from the literature.

Theorem 2. *(Davidson & Szarek (2001), Theorem II.13)*

Given $n, p \in \mathbb{N}$, with $p \leq n$, let Γ be a $p \times n$ matrix with i.i.d. drawn from a normal distribution with mean 0 and variance $1/n$. Then the largest and smallest singular values of Γ, $\sigma_1(\Gamma)$ and $\sigma_p(\Gamma)$, are such that

$$\max\left(\mathbb{P}\left(\sigma_1(\Gamma) \geq 1 + \sqrt{\frac{p}{n}} + t\right), \mathbb{P}\left(\sigma_p(\Gamma) \leq 1 - \sqrt{\frac{p}{n}} - t\right)\right) \leq \exp\left(-\frac{nt^2}{2}\right),$$

for $t > 0$.

Our goal is to bound the spectral norm of the error terms:

$$E^n = \begin{pmatrix} \Sigma_X^n \\ 2\Sigma_{ZX}^n \end{pmatrix} - \mathcal{F}_{\Sigma_Z^n}(K^*).$$

We recall the notations defined in Proposition 5: we write $\psi_Z \triangleq ||\Sigma_Z^n||_2$, $\psi_X^* \triangleq ||K_X^{*-1}||_2$ and $\phi_{ZX}^* \triangleq ||K_{ZX}^*||_2$.

Before proving a result about $||E^n||_2$, we start by deriving bounds on $||E_{ZX}^n||_2$ and $||E_X^n||_2$ independently.

Proposition 10. *Let $||E_{ZX}^n||_2$ be defined as above. If Σ_Z^n is non-singular, then for any $\delta > 0$ and $n \geq \frac{16\psi_Z\psi_X^*}{\delta^2}(\sqrt{m} + \sqrt{p})^2$ we have*

$$\mathbb{P}\left(||E_{ZX}^n||_2 \geq \delta\right) \leq \exp\left(-\frac{n\delta^2}{32\psi_Z\psi_X^*}\right).$$

Proof. Recall that E_{ZX}^n was defined as:

$$E_{ZX}^n = 2\left(\Sigma_{ZX}^n + \Sigma_Z K_{ZX}^* K_X^{*-1}\right).$$

Recall also that the samples $(Y_X)_i$ are drawn conditionally on $(Y_Z)_i$ according to:

$$(Y_X)_i | (Y_Z)_i \sim \mathcal{N}\left(-K_X^{*-1} K_{ZX}^{*T} (Y_Z)_i, K_X^{*-1}\right),$$

so that

$$Y_X = -Y_Z K^*_{ZX} K^{*-1}_X + R$$

, with R a matrix whose rows are drawn according to $R_i \sim \mathcal{N}(0, K^{*-1}_X), \forall 1 \leq i \leq n$. It follows that

$$\frac{n}{2} E^n_{ZX} = Y_Z^T Y_X + Y_Z^T Y_Z K^*_{ZX} K^{*-1}_X = Y_Z^T R.$$

Thus, bounding $||E^n_{ZX}||_2$ amounts to bounding the largest singular value of $\frac{2}{n} Y_Z^T R$.

Following the arguments of Lemma 4 in Yin & Li (2011), we have that $\frac{1}{\sqrt{n}} Y_Z^T R K^{*\,1/2}_X$ is an $m \times p$ matrix whose rows are uniformly distributed according to the $m \times 1$ multivariate normal distribution $\mathcal{N}(0, \frac{1}{n} Y_Z^T Y_Z)$. Therefore,

$$\Gamma := \frac{1}{\sqrt{n}} \Sigma^{n\,-1/2}_Z Y_Z^T R K^{*\,1/2}_X$$

is a random $m \times p$ matrix whose entries are i.i.d. drawn from the $\mathcal{N}(0, 1)$ distribution. We write $p_+ := max(m, p)$ and $p_- := min(m, p)$. Now, we have

$$\begin{aligned}
\mathbb{P}\left(||E^n_{ZX}||_2 \geq \delta\right) &= \mathbb{P}\left(||\frac{2}{n} Y_Z^T R||_2 \geq \delta\right) \\
&= \mathbb{P}\left(\frac{2}{\sqrt{n}} ||\frac{1}{\sqrt{n}} \Sigma^{n\,1/2}_Z \Sigma^{n\,-1/2}_Z Y_Z^T R K^{*1/2}_X K^{*-1/2}_X||_2 \geq \delta\right) \\
&\leq \mathbb{P}\left(\frac{2\sqrt{\psi_Z \psi^*_X}}{\sqrt{n}} ||\frac{1}{\sqrt{n}} \Sigma^{n\,-1/2}_Z Y_Z^T R K^{*1/2}_X||_2 \geq \delta\right) \\
&= \mathbb{P}\left(\frac{2\sqrt{p_+ \psi_Z \psi^*_X}}{\sqrt{n}} ||\frac{1}{\sqrt{p_+}} \Gamma||_2 \geq \delta\right) \\
&= \mathbb{P}\left(||\frac{1}{\sqrt{p_+}} \Gamma||_2 \geq \frac{\delta\sqrt{n}}{2\sqrt{p_+ \psi_Z \psi^*_X}}\right).
\end{aligned}$$

The $m \times p$ matrix $\frac{1}{\sqrt{p_+}} \Gamma$ satisfies Theorem 2. Applying it, we obtain:

$$\mathbb{P}\left(||\frac{1}{\sqrt{p_+}} \Gamma||_2 \geq 1 + \sqrt{\frac{p_-}{p_+}} + t\right) \leq \exp\left(-\frac{p_+ t^2}{2}\right),$$

so that

$$\mathbb{P}\left(||E^n_{ZX}||_2 \geq \frac{2\sqrt{\psi_Z \psi^*_X p_+}}{\sqrt{n}} + \frac{2\sqrt{\psi_Z \psi^*_X p_-}}{\sqrt{n}} + t\right) \leq \exp\left(-\frac{nt^2}{8\psi_Z\psi^*_X}\right).$$

By our assumptions on n, we know that $\frac{\delta}{2} \geq \frac{2\sqrt{\psi_Z\psi^*_X}}{\sqrt{n}}(\sqrt{p_-} + \sqrt{p_+})$. We conclude the proof by picking $t = \delta/2$. □

We now turn to $||E^n_X||_2$ and prove a similar result.

Proposition 11. *(see Chandrasekaran et al. (2012), suppl. materials Lemma 3.9) Let E^n_X be defined as above. Given any $\delta > 0$, with $\delta \leq 8\psi^*_X$ and $n \geq \frac{64p\psi^{*2}_X}{\delta^2}$. Then,*

$$\mathbb{P}\,||E^n_X||_2 \geq \delta) \leq 2\exp\left(-\frac{n\delta^2}{128\psi^{*2}_X}\right).$$

Proof. We have:

$$E^n_X = \Sigma^n_X - (K^{*-1}_X + K^{*-1}_X K^{*T}_{ZX} \Sigma^n_Z K^*_{ZX} K^{*-1}_X).$$

Just like in the previous proposition, we can rewrite the error term in terms of R, an $n \times p$ matrix whose rows are drawn from $\mathcal{N}(0, K^{*-1}_X)$:

$$E^n_X = \frac{1}{n} R^T R - K^{*-1}_X.$$

The rest of the proof follows a progression which is identical to Chandrasekaran et al. (2012) (suppl. materials, Lemma 3.9). □

We now put these propositions together in order to bound $||E^n||_2$.

Proposition 12. *Let E^n be defined as above. For any $\delta > 0$ with $\delta \leq 8\sqrt{2}\psi^*_X$, let $n \geq \frac{1}{\delta^2}\max(128p\psi^{*2}_X, 32\psi_Z\psi^*_X(\sqrt{m} + \sqrt{p})^2)$. Then,*

$$\mathbb{P}\left(||E^n||_2 \geq \delta\right) \leq \max\left(2\exp\left(-\frac{n\delta^2}{256\psi^{*2}_X}\right), \exp\left(-\frac{n\delta^2}{64\psi_Z\psi^*_X}\right)\right).$$

Proof. We know that

$$\max(||E_X^n||_2, ||E_{ZX}^n||_2) \leq ||E^n||_2 \leq \sqrt{2}\max(||E_X^n||_2, ||E_{ZX}^n||_2)$$

so that

$$\mathbb{P}(||E^n||_2 \geq \delta) \leq \max\left(\mathbb{P}(\sqrt{2}||E_X^n||_2 \geq \delta), \mathbb{P}(\sqrt{2}||E_{ZX}^n||_2 \geq \delta)\right).$$

From Proposition 10, we have

$$\mathbb{P}(\sqrt{2}||E_{ZX}^n||_2 \geq \delta) \leq \exp\left(-\frac{n\delta^2}{64\psi_Z\psi_X^*}\right),$$

for $n \geq \frac{32\psi_Z\psi_X^*}{\delta^2}(\sqrt{m}+\sqrt{p})^2$.

Applying Proposition 11, we have for $\delta \leq 8\sqrt{2}\psi_X^*$ and $n \geq \frac{128p\psi_X^{*2}}{\delta^2}$:

$$\mathbb{P}(\sqrt{2}||E_X^n||_2 \geq \delta) \leq 2\exp\left(-\frac{n\delta^2}{256\psi_X^{*2}}\right).$$

□

Finally, we can prove a result that allows us to condition on the norm of the error terms as a function of the number of samples and the dimensions of the problem, p and m.

Corollary 5. *Let* $M = \max\left(1, \frac{\psi_Z}{4\psi_X^*}(1+\sqrt{\frac{m}{p}})^2\right)$. *Let* $\delta_n = \sqrt{\frac{256\psi_X^{*2}pM}{n}}$. *Then for* $n \geq 2pM$*,*

$$\mathbb{P}\left(\|E^n\|_2 \leq \delta_n\right) \geq 1 - \max\left(2\exp\left(-pM\right), \exp\left(-\frac{4\psi_X^*}{\psi_Z}pM\right)\right).$$

Proof. We show that

$$\mathbb{P}\left(||E^n||_2 \geq \delta_n\right) \leq \max(2\exp(-pM), \exp(-\frac{4\psi_X^*}{\psi_Z}pM)).$$

First of all, remark that for $n \geq 2pM$, we have $\delta_n \leq 8\sqrt{2}\psi_X^*$, so we can apply Proposition 12.

Then, by Proposition 12, we have a bound of the form

$$\mathbb{P}\left(||E^n||_2 \geq \delta_n\right) \leq \max\left(2\exp\left(-A\right), \exp\left(-B\right)\right),$$

with

$$A = \frac{n}{256\psi_X^{*2}} \frac{256\psi_X^{*2} pM}{n} = pM,$$

and

$$B = \frac{n}{64\psi_Z \psi_X^*} \frac{256\psi_X^{*2} pM}{n} = \frac{4\psi_X^*}{\psi_Z} pM = \frac{4\psi_X^*}{\psi_Z} pM.$$

□

2.5.7 Proof of consistency

Before concluding with our final result, the consistency of our estimator, we state a proposition which summarises the results of the previous sections, namely Sections 2.5.2, 2.5.3, 2.5.4 and 2.5.5.

Proposition 13. *(Summary of the previous results)*
Define the following constants,

$$C_1 = \frac{48}{\alpha} + \frac{1}{\psi_X^{*2}\left(1 + 2\frac{\psi_Z}{\psi_X^*}\left(1 + \psi_X^* \phi_{ZX}^*\right)^2\right)};$$

$$C_2 = \left(1 + \frac{24(2-\nu)}{\nu}\right) C_1^2 D \psi_X^{*2} \left(1 + 2\frac{\psi_Z}{\psi_X^*}\left(1 + \psi_X^* \phi_{ZX}^*\right)^2\right);$$

$$C_3 = C_1 + \frac{3\alpha C_1^2 (2-\nu)}{4(3-\nu)}; \; C_4 = \max\{C_2, C_3\}; \; C_5 = \frac{C_1 \nu \alpha}{\beta(2-\nu)};$$

$$\sigma = \textit{smallest non-zero singular value of } L^*;$$

$$\theta = \textit{smallest entry in magnitude of } S^*.$$

Assume that γ is in the acceptable range, i.e.

$$\gamma \in \left[\frac{3\xi(T)\beta(2-\nu)}{\nu\alpha}, \frac{\nu\alpha}{2\mu(\Omega)\beta(2-\nu)}\right].$$

Assume that $\sigma \geq \frac{C_4\lambda_n}{\xi(T)^2}$ and $\theta \geq \frac{C_5\lambda_n}{\mu(\Omega)}$. Further assume that $g_\gamma(\mathcal{A}^\dagger E^n) \leq \frac{\lambda_n\nu}{6(2-\nu)}$ and

$$\lambda_n \leq \frac{3\alpha(2-\nu)}{16(3-\nu)}\frac{1}{1+\frac{\alpha}{6\beta}}\min\left(\frac{1}{4\psi_X^*}, \frac{\alpha\xi(T)}{128D\psi_X^*\psi^2(1+\frac{\alpha}{6\beta})}\right).$$

Then, there exists a T' and a corresponding unique solution $(\hat{S}_\Omega, \hat{L}_{T'})$ to (2.3) with the following properties:

1. *$sign(\hat{S}_\Omega) = sign(S^*)$, $rank(\hat{L}_{T'}) = rank(L^*)$ and $(\hat{L}_{T'})_X \succeq 0$. Moreover, $T(\hat{L}_{T'}) = T'$ and $\rho(T,T') \leq \frac{8\xi(T)}{73} \leq \frac{\xi(T)}{4}$.*

2. *As before, write $\mathcal{C}_{T'} = \mathcal{P}_{T'^\perp}(L^*)$. Then we have $g_\gamma(\mathcal{A}^\dagger\mathcal{I}^*_{\Sigma^n_Z}\mathcal{C}_{T'}) \leq \frac{\lambda_n\nu}{6(2-\nu)}$ and $||\mathcal{C}_{T'}||_2 \leq \frac{16(3-\nu)\lambda_n}{3\alpha(2-\nu)}$.*

In addition, if

$$g_\gamma(\mathcal{A}^\dagger R_{K^*}\mathcal{A}(\Delta_S, \Delta_L)) \leq \frac{\lambda_n\nu}{6(2-\nu)},$$

then $(\hat{S}_\Omega, \hat{L}_{T'}) = (\hat{S}, \hat{L})$ is the unique solution to the original problem (including the constraint $L_X \succeq 0$).

Our goal is now to combine the results of the previous section (bound on the error terms) in order to show under which conditions the assumptions of Proposition 13 are satisfied. We use Proposition 13 and Corollary 5.

We reuse the notations and constants introduced throughout the proof and refer the reader to the statement of Theorem 1 for a list of their definitions.

We define the list of assumptions required to achieve algebraic consistency.

Assumption 3. *(Assumptions for Algebraic Consistency)*

1. *Set*
$$C_6 = \frac{\alpha\nu}{32(3-\nu)D}\frac{1}{1+\frac{\alpha}{6\beta}}\min\left(\frac{1}{4\psi_X^*}, \frac{\alpha\xi(T)}{512D\psi_X^*\psi^2(1+\frac{\alpha}{6\beta})}\right)$$
and, for M defined as in Corollary 5., let n be such that
$$n \geq \frac{pM}{\xi(T)^4}\max(2, \frac{256\psi_X^{*2}}{C_6^2}).$$

2. *For M defined as before, set*
$$\delta_n = \sqrt{\frac{256\psi_X^{*2}pM}{n}},$$
and let
$$\lambda_n = \frac{6D\delta_n(2-\nu)}{\xi(T)\nu}.$$

3. *Let the minimum non-zero singular value of L^* be such that*
$$\sigma \geq \frac{C_4\lambda_n}{\xi(T)^2}.$$

4. *Let the minimum magnitude nonzero entry θ of S^* be such that*
$$\theta \geq \frac{C_5\lambda_n}{\mu(\Omega)}.$$

Assuming that Assumption 3 holds, the proof of our main theorem (which is restated for convenience in Theorem 1) is in all points identical to the proof given in Chandrasekaran et al. (2012), Section 5.5. We refer the reader to their proof for the details. In particular, the proof makes use of Proposition 6 in order to show that $g_\gamma(\Delta_S, \Delta_L) \leq \frac{32(3-\nu)\lambda_n}{3\alpha(2-\nu)}$.

2.6 A note on the validity of these results for arbitrary matrices

Throughout our proof, we relied on some concepts and intermediate results that were initially proven in Chandrasekaran et al. (2012) where it is assumed that both S and L are positive (semi-)definite matrices, or at least admit an eigenvalue decomposition over $\mathbb{R}$. Whenever it was necessary, we proved new results that are valid for arbitrary matrices (*e.g.* the results pertaining to the curvature of the rank variety in Section 2.4.1). There are cases, however, where we used the results of Chandrasekaran et al. (2012) directly and the reader might wonder why those hold for arbitrary matrices and whether their proofs carry over from the special case of square matrices. Here, we provide more details on this question.

First, it should be noted that many key definitions were initially published in Chandrasekaran et al. (2009), which itself deals with *arbitrary* matrices. In particular, the definitions of $\xi(T(M)$ and $\mu(\Omega(M))$ are valid for any matrix M, along with the concepts that come with them: variety of sparse and low-rank matrices, expression of the tangent spaces at any smooth point, etc... Moreover, it is clear that the definition of $\rho(\cdot,\cdot)$ (Equation (2.1)) is also valid for arbitrary subspaces T_1, T_2 since it is simply the $||\cdot||_{2\to 2}$ operator norm of some orthogonal projector.

The critical point that needs addressing concerns the applicability of Proposition 1 which is invoked time and again in the consistency proof and is the only Proposition that we reuse directly from Chandrasekaran et al. (2012) (where it is called Proposition 3.3). First, we see that the only dependence of Proposition 1 on the log-likelihood comes from the Fisher Information Matrix $\mathcal{I}^*_{\Sigma^n_Z}$. However, its specific form is irrelevant: all the important features of the FIM are captured by α, β, δ and ν. These quantities are well-defined and behave in the same way for any log-likelihood. Second, the reader can check that the proof of Proposition 3.3 (in Chandrasekaran et al. (2012)) relies on two Lemmas (3.1 and 3.2)

which involve only elementary properties of ρ, ξ and μ, none of which require the matrices at hand to have any particular structure. In conclusion, Proposition 1 (and its proof) is "likelihood agnostic" and is more concerned about the fundamental problem of decomposing a given matrix into its summands. This is a problem which has been tackled for arbitrary matrices using the exact same apparatus in Chandrasekaran et al. (2009) and it is therefore not surprising to see that the proof techniques for identifiability found in Chandrasekaran et al. (2012) carry over straightforwardly to our setting.

Finally, let us conclude by pointing out that our considering arbitrary matrices is not the only source of variation between our results and those given in Chandrasekaran et al. (2012). The log-likelihood – via its second and third derivatives – is also very different and introduces most of the complications.

3 Optimisation: Supplementary Materials for Section 4

In Section 3, two approaches are suggested to optimise our objective function

$$(\hat{S}_X, \hat{L}_X, \hat{S}_{ZX}, \hat{L}_{ZX}) = \\ \underset{S_X, L_X \in \mathbb{R}^{p\times p}; S_{ZX}, L_{ZX} \in \mathbb{R}^{m\times p}}{\arg\min} -\ell(S_X, L_X, S_{ZX}, L_{ZX}; \Sigma_Z^n, \Sigma_X^n, \Sigma_{ZX}^n) + \lambda_n(\gamma \|S\|_1 + \|L\|_*) \\ \text{s.t. } S_X - L_X \succ 0, L_X \succeq 0 \text{ and } S = \begin{pmatrix} S_X \\ S_{ZX} \end{pmatrix}, L = \begin{pmatrix} L_X \\ L_{ZX} \end{pmatrix}, \quad (3.1)$$

where the log-likelihood is expressed in terms of the sample covariances Σ_Z^n, Σ_X^n and Σ_{ZX}^n:

$$\ell(S_X, L_X, S_{ZX}, L_{ZX}; \Sigma_Z^n, \Sigma_X^n, \Sigma_{ZX}^n) = \log\det(S_X - L_X) - Tr\left(\Sigma_X^n(S_X - L_X)\right) - \\ 2Tr\left(\Sigma_{ZX}^n(S_{ZX} - L_{ZX})^T\right) - Tr\left(((S_X - L_X)^{-1}(S_{ZX} - L_{ZX})^T\Sigma_Z^n(S_{ZX} - L_{ZX})\right). \quad (3.2)$$

We suggest fitting the model using either the Alternative Direction Method of Multipliers (ADMM) (Boyd 2011) or a Semi-Definite Programming approach (SDP) (Vandenberghe & Boyd 1996, Wang et al. 2010, Tütüncü et al. 2003). We choose to describe (and implement) both approaches because they have different strengths. Our ADMM implementation is fast but tailored to the problem at hand, which makes it hard for the reader to modify the model. On the other hand, SDP solvers might not be as fast but implementing new problems is very easy thanks to software packages such as YALMIP (Löfberg 2004). Both implementations are made available with this paper.

3.1 A three-block Alternative Direction Method of Multipliers (ADMM)

3.1.1 The Alternative Direction Method of Multipliers

The ADMM is a first-order method which is well-suited to the optimisation of objective functions expressed as the sum of n separable closed convex functions, under some linear constraints,*i.e.*:

$$\begin{gathered}\underset{x_1,x_2,\ldots}{\arg\min}\; f_1(x_1) + f_2(x_2) + \ldots + f_n(x_n) \\ \text{such that } A_1x_1 + A_2x_2 + \ldots + A_nx_n = b \\ \text{and } x_i \in \mathcal{C}_i,\ \forall i \in \{1, \ldots, n\},\end{gathered}$$

where the A_i are linear operators and the $\mathcal{C}_i$ are closed convex sets. In order to enforce the constraints, the ADMM resorts to an augmented Lagrangian defined as

$$\mathcal{L}_\mu(x_1, \ldots; U) := f_1(x_1) + \ldots + f_n(x_n) + \frac{\mu}{2}\left\| A_1x_1 + \ldots A_nx_n - b + \frac{U}{\mu} \right\|_F^2,$$

with $\mu > 0$ a penalty parameter and $\|\cdot\|_F$ the Frobenius norm. Usually, optimising $\mathcal{L}_\mu$ directly is not possible. Instead, ADMM conveniently decomposes the problem into subproblems which are solved iteratively. Given the parameters from iteration k, $(x_1^k, \ldots, x_n^k, U^k)$, one computes

$$\begin{aligned}
x_1^{k+1} &:= \arg\min_{a \in \mathcal{C}_1} \mathcal{L}_\mu(a, x_2^k, \ldots, x_n^k; U^k) \\
x_2^{k+1} &:= \arg\min_{a \in \mathcal{C}_2} \mathcal{L}_\mu(x_1^{k+1}, a, \ldots, x_n^k; U^k) \\
&\vdots \\
x_n^{k+1} &:= \arg\min_{a \in \mathcal{C}_n} \mathcal{L}_\mu(x_1^{k+1}, \ldots, a; U^k) \\
U^{k+1} &:= U^k + \mu \left(A_1^{k+1} x_1 + \ldots A_n^{k+1} x_n - b \right).
\end{aligned}$$

In the special case $n = 2$, ADMM has been extensively studied in the literature and its global convergence proven (see Boyd et al. (2010) and references therein for a thorough account of ADMM's theoretical and practical properties). For our purposes, we draw the attention of the reader to a few points outlined in Boyd et al. (2010):

- ADMM usually converges to a reasonable accuracy within a few tens of iterations, which is enough for most statistical applications (including ours). Should a very high accuracy be required, second-order methods might be preferable. An efficient strategy is to first use ADMM in order to obtain an approximate solution and then to use this solution as a starting point for a second-order algorithm, as in the SDPNAL+ solver of Yang et al. (2015).

- The updates of x_i^{k+1} need not be computed exactly for ADMM to converge. Thus, the x_i-minimisation steps can be carried out with iterative solvers (Boyd 2011, Section 4.3.1).

- To achieve a good convergence rate, the accuracy to which the updates are computed ought to increase at each iteration. If an iterative algorithm is used to compute an approximate solution to any of the x_i^{k+1} updates, a possible strategy is to gradually increase the accuracy at each iteration. To keep the cost of each iteration under control, one can terminate the iterative procedure early if the required accuracy is not reached.

- Since the sequence $x_i^0, x_i^1, \ldots, x_i^k$ is converging, one can greatly benefit from initialising the iterative algorithm with the solution from the previous iteration, *i.e.* warm-starting the iterative procedure used to compute x_i^{k+1} with x_i^k. This makes it possible to compute x_i^{k+1} more and more accurately as k increases without increasing the computational cost.

When the number of blocks, n, is greater than two, the convergence of ADMM is not guaranteed anymore (Chen et al. 2016). Recently, convergence results have been established in a number of special cases, including the case where ∇f_N is Lipschitz continuous and $A_n = I$ (the identity matrix) (Lin et al. 2016). Problems for which A_n are called *sharing problems* and are very common in statistics. The problem at hand, problem (3.1) can be recast in that framework, which justifies the use of ADMM in our context.

3.1.2 Writing the updates of ADMM for Problem (3.1)

We now show that our objective function is a sharing problem with three blocks, which justifies the use of ADMM to obtain solutions.

In our setting, some parameters are non-square matrices (*i.e.* S and L) whose upper-parts are required to be positive-definite matrices. In order to avoid cluttered notations,

we introduce the set:

$$\mathcal{C}^{\succ} := \{M \text{ such that } M = \begin{pmatrix} M_X \\ M_{ZX} \end{pmatrix}, M_X \in \mathbb{R}^{p\times p}, M_{ZX} \in \mathbb{R}^{m\times p}, M_X \succ 0\},$$

that is, the convex set of all $(p+m)\times p$ matrices whose first p rows form a positive definite matrix. Likewise, $\mathcal{C}^{\succeq}$ is the set of matrices whose first p rows form a positive semi-definite matrix. We write $\mathcal{I}_{\mathcal{C}^{\succ}}$ for the indicator function of the set $\mathcal{C}^{\succ}$.

Using this notation, we can rewrite (3.1) as a sharing problem with three separable convex functions. Problem (3.1) is equivalent to

$$\begin{aligned} &\underset{A,S,L}{\arg\min} \quad -\ell(A;\Sigma_X^n,\Sigma_Z^n,\Sigma_{ZX}^n) + \lambda_n(\|S\|_1 + \|L\|_*) \\ &\text{such that } A - S + L = 0 \text{ and } L \in \mathcal{C}^{\succeq}. \end{aligned} \tag{3.3}$$

By setting $f_1 = \lambda_n\gamma||\cdot||_1$, $f_2 = \lambda_n||\cdot||_*$ and $f_3 = -\ell$, we recover the sharing problem with $n = 3$ described in the previous section. In particular, f_3 is smooth (a fortiori, ∇f_3 is Lipschitz continuous) and $A_3 = I$ so that ADMM is guaranteed to converge, by (Lin et al. 2016, Th 4.5)

Following the description of ADMM given above, the updates for the parameters A, S, L and the Lagrange multiplier U are given by:

$$A^{k+1} := \text{prox}_{(-\ell(\cdot)+\mathcal{I}_{\mathcal{C}^{\succ}}(\cdot))/\mu}(S^k + L^k + \frac{U^k}{\mu}), \tag{3.4}$$

$$S^{k+1} := \text{prox}_{\lambda_n\gamma||\cdot||_1/\mu}(A^{k+1} + L^k + \frac{U^k}{\mu}), \tag{3.5}$$

$$L^{k+1} := \text{prox}_{(\lambda_n||\cdot||_*+\mathcal{I}_{\mathcal{C}^{\succeq}}(\cdot))/\mu}(S^{k+1} - A^{k+1} - \frac{U^k}{\mu}), \tag{3.6}$$

$$U^{k+1} := U^k + \mu(A^{k+1} - S^{k+1} + L^{k+1}), \tag{3.7}$$

where we recall that all parameters (A, S, L, U) are of size $(p+m)\times p$. Here, we have introduced the *proximal operator* $\text{prox}_{f/\kappa}$ of a function $f : \mathbb{R}^{p_1\times p_2} \to \mathbb{R}\cup\{+\infty\}$ which is

defined for some $\kappa > 0$, as

$$\text{prox}_{f/\kappa}(V) := \arg\min_X f(X) + \frac{\kappa}{2}||X - V||_F^2.$$

Due to their prominent role in solving regularised MLEs, the proximal operators of many common functions have been investigated in detail (Parikh & Boyd 2014). Here, we will need the following operators (see Parikh & Boyd (2014) for more examples):

- $f(M) := ||M||_1$, for some arbitrary matrix $M \in \mathbb{R}^{p_1 \times p_2}$. Then, the $(i, j)^{th}$ entry of the proximal operator is given by:

  $$(\text{prox}_{f/\mu}(M))_{ij} := \text{sign}(M_{ij}) \max(0, (|M_{ij}| - \frac{1}{\mu})).$$

  This is the so-called "soft-thesholding" operator.

- $f := \mathcal{I}_C$, the indicator function of some convex set C. Then, we have:

  $$\text{prox}_{f/\mu}(M) := \arg\min_{X \in C} ||X - M||_F^2,$$

  this is the projection of M onto C. In particular, if C is the set of positive-definite matrices, then any matrix which admits an eigenvalue decomposition over $\mathbb{R}$ is projected onto C by setting its negative eigenvalues to 0.

- $f(M) := ||M||_*$, for some arbitrary matrix $M \in \mathbb{R}^{p_1 \times p_2}$. Letting $M = U\Sigma V^T$ be the singular value decomposition of M, the proximal operator of the nuclear norm is given by:

  $$\text{prox}_{f_\mu}(M) := U\text{diag}(\max(0, \Sigma_{ii} - \frac{1}{\mu}))V^T,$$

  where $\text{diag}(a_{ii})$ is the matrix obtained by setting the ith entry along the diagonal to a_{ii} and everything else to 0.

We now see that updates (3.5) and (3.7) can be computed very efficiently and in closed-form. In particular, (3.5) is the proximal operator of the ℓ_1-norm and is computed with the soft-thresholding operator.

(3.4) cannot be computed in closed-form but it involves a smooth function which makes obtaining an approximation to A^{k+1} straightforward. In our implementation, we use an accelerated gradient descent algorithm in order to approximate A^{k+1}. In practice, the value from the previous iteration (A^k) is used as initial parameter and we found that, after the first few updates of A^k, only handful of iterations are necessary to reach termination (typically less than 10). If termination is not reached after a fixed number of iterations (in our case 10), we exit early.

3.1.3 Computation of the proximal operator (3.6)

We now turn to (3.6) which does not admit a closed-form solution and we suggest an iterative splitting algorithm to solve this problem.

For clarity, let us unfold the definition of (3.6) and write the objective function explicitly. Problem (3.6), is rewritten as

$$(L_X^{k+1}, L_{ZX}^{k+1}) :=$$
$$\underset{L_X \in \mathbb{R}^{p\times p}, L_{ZX}\mathbb{R}^{m\times p}}{\arg\min} \quad \lambda_n \left\| \begin{pmatrix} L_X \\ L_{ZX} \end{pmatrix} \right\|_* + \mathcal{I}_{\mathcal{C}^{\succeq}}\left(\begin{pmatrix} L_X \\ L_{ZX} \end{pmatrix} \right) + \frac{\mu}{2} \left\| \begin{pmatrix} S_X^{k+1} - A_X^{k+1} + U_X^k/\mu \\ S_{ZX}^{k+1} - A_{ZX}^{k+1} - U_{ZX}^k/\mu \end{pmatrix} \right\|_F^2 .$$

This update (3.6) requires the computation of the proximal operator of $\lambda_n||\cdot||_* + \mathcal{I}_{\mathcal{C}^{\succeq}}(\cdot)$. Considered independently, these proximal operators admit well-known closed form solutions that rely on the thresholding of the eigenvalues of L_X and the singular values of L, respectively (see Section 3.1.2 or Parikh & Boyd (2014)). The proximal operator of their sum, however, cannot be computed in closed form. We must resort to the "Dykstra-like" proximal

algorithm of Combettes & Pesquet (2009) which generates a sequence that converges to the solution of (3.6) (Combettes & Pesquet 2009, Proposition 5.3). See Algorithm 1 below for more details. Note that Algorithm 1 is itself a splitting algorithm of the same family as ADMM and enjoys similar computational properties (*e.g.* convergence to a reasonable accuracy after very few iterations, as explained in Boyd et al. (2010) and (Goldstein & Osher 2009, Suppl. Mat.).

Initialisation: Set $X_0 = S^{k+1} - A^{k+1} - \frac{U^k}{\mu}$, $P_0 = 0$, $Q_0 = 0$;

for $k = 1, \ldots, K$ **do**

  $Y_k = \text{prox}_{\mathcal{I}\mathcal{C}^{\succeq}}(X_k + P_k)$;

  $P_{k+1} = X_k + P_k - Q_k$;

  $X_{k+1} = \text{prox}_{\lambda_n \|\cdot\|_* / \mu}(Y_k + Q_k)$;

  $Q_{k+1} = Y_k + Q_k - X_{k+1}$

end

Result: X_k.

Algorithm 1: "Dykstra-like" algorithm of Combettes & Pesquet (2009). The sequence X_k converges to the solution of (3.6). In practice, $\frac{\|X_{k+1}-X_k\|_F}{\|X_k\|_F} \leq \epsilon$ is used as a stopping criterion. We choose a fixed ϵ of 10^{-10}.

3.1.4 Summary

We have derived the updates of a 3-block ADMM that provably converges to the solution of (3.1). We iteratively solve four subproblems ((3.4) - (3.7)). An approximate solution to two of these of these problems is obtained thanks to a few iterations of some sub-algorithm (*i.e.* gradient descent for (3.4) and Dysktra-like algorithm for (3.6)). Each iteration of ADMM is dominated by the computation of A^{k+1} (with a cost of $\mathcal{O}(p^3)$) and L^{k+1} (with a cost of $\mathcal{O}(\max(p^3, pm^2, p^2m))$, due to the SVD).

The authors of Ye et al. (2011) and Ma et al. (2013) derived similar updates for ADMM in order to obtain solutions for the Low-Rank plus Sparse (LR+S) estimator of Chandrasekaran et al. (2012), with the difference that all updates could be computed in closed-form. In particular, in the case of LR+S, L is a $(p+m) \times (p+m)$ positive semi-definite matrix and the proximal operator $||L||_*$ can be computed in closed-form. One iteration of this ADMM algorithm used to solve LR+S has time complexity $\mathcal{O}((p+m)^3)$.

3.2 Semi-Definite Programming

The solvers made available in the MATLAB® packages SDPT3 and Logdet-PPA are capable of solving problems of the form (Tütüncü et al. 2003, Wang et al. 2010):

$$\underset{X_1, X_2, \ldots}{\arg\min} \; Tr(X_1 C_1^T) + Tr(X_2 C_2^T) + \ldots + a_1 \log\det(X_1) \tag{3.8}$$

subject to a number of linear, quadratic and positive semidefinite constraints [1]. Our goal is then to recast (3.1) as a problem of the same form as (3.8). This involves introducing additional constrained variables, as shown below.

First, we focus on the form taken by the likelihood of a Gaussian conditional random field (GCRF). Estimating a GCRF amounts to solving

$$\underset{K_X, K_{ZX}}{\arg\min} - \log\det K_X + Tr(\Sigma_X^n K_X + 2K_{ZX}^T \Sigma_{ZX}^n + K_{ZX} K_X^{-1} K_{ZX}^T \Sigma_Z^n) \tag{3.9}$$

with $K_X \succ 0$. This differs from (3.8) by the presence of the term $K_{ZX} K_X^{-1} K_{ZX}^T$ which involves the inverse of the parameter K_X. To address this problem, we introduce a new variable W and solve the equivalent problem

$$\underset{K_X, K_{ZX}}{\arg\min} = - \log\det K_X + Tr(K \Sigma_O^n)$$

[1]See references for a formulation of the problem in its full generality.

such that

$$K = \begin{pmatrix} W & K_{ZX} \\ K_{XZ} & K_X \end{pmatrix} \succeq 0, \text{ and } K_X \succ 0.$$

This formulation is equivalent to (3.9) because Σ_Z^n is a covariance matrix and therefore positive-semidefinite. As a result, the constraint $K \succeq 0$ is equivalent to $W = K_{ZX} K_X^{-1} K_{ZX}^T$ [2].

Having addressed this issue, there remains the problem of recasting the penalty $\lambda_n(\gamma \|S\|_1 + \|L\|_*)$ as a combination of linear constraints and trace terms. One shows that (Chandrasekaran et al. 2009):

$$\arg\min_{S,L} \gamma \|S\|_1 + \|L\|_*$$

is equivalent to

$$\arg\min_{S,L,F,H_1,H_2} \lambda_n \left(\gamma \mathbf{1}^T F \mathbf{1} + \frac{1}{2} \left(Tr(H_1) + Tr(H_2) \right) \right)$$

subject to

$$\begin{pmatrix} H_1 & L \\ L^T & H_2 \end{pmatrix} \succeq 0, \quad -F_{ij} \leq S_{ij} \leq F_{ij}, \ \forall i,j;$$

where the latter constraint is to be understood elementwise.

Putting everything together, we obtain the following SDP formulation of (3.1):

$$\arg\min_{S_X,L_X,S_{ZX},L_{ZX},W,F,H_1,H_2} Tr(K\Sigma_O^n) - \log\det S_X + \lambda_n \left(\gamma \mathbf{1}^T F \mathbf{1} + \frac{1}{2} \left(Tr(H_1) + Tr(H_2) \right) \right)$$

$$\text{subject to } K \succeq 0, \ S_X \succ 0, \ L_X \succeq 0, \ \begin{pmatrix} H_1 & L \\ L^T & H_2 \end{pmatrix} \succeq 0, \quad -F_{ij} \leq S_{ij} \leq F_{ij}, \ \forall i,j;$$

$$\text{where } K = \begin{pmatrix} W & S_{ZX} - L_{ZX} \\ S_{ZX}^T - L_{ZX}^T & S_X - L_X \end{pmatrix}, \ S = \begin{pmatrix} S_X \\ S_{ZX} \end{pmatrix}, \ L = \begin{pmatrix} L_X \\ L_{ZX} \end{pmatrix}. \quad (3.10)$$

[2]We thank Prof. Defeng Sun for his help on this problem.

Problem (3.10) can then easily be implemented using *e.g.* YALMIP and solved using LogdetPPA or SDPT3 (Löfberg 2004, Wang et al. 2010, Tütüncü et al. 2003).

4 Simulations: Supplementary Materials for Section 5

4.1 Description of the graphical models

In the interest of space, Section 5 of the main paper did not contain details about the distribution of the parameters in our simulations. We give more details here. The code used to generate our synthetic data is also made available with the paper.

Recall that for all simulations, each observation is generated according to a model of the form

$$\begin{pmatrix} Y_X \\ Y_H \end{pmatrix} | Y_Z \sim \mathcal{N} \left\{ - \begin{pmatrix} S_X^* & M_{XH}^* \\ {M_{XH}^*}^T & M_H^* \end{pmatrix}^{-1} \begin{pmatrix} {M_{ZX}^*}^T \\ 0 \end{pmatrix} Y_Z, \begin{pmatrix} S_X^* & M_{XH}^* \\ {M_{XH}^*}^T & M_H^* \end{pmatrix}^{-1} \right\},$$

with Y_Z a random vector of size p whose entries are drawn independently from a t-distribution with 4 degrees of freedom. Y_X is also of size p. Here, Y_X and Y_H are drawn jointly from a conditional random Markov field but only Y_X and Y_Z are observed, which implies that $L_X^* = M_{XH}^* {M_H^*}^{-1} {M_{XH}^*}^T$. The matrices S_X^*, L_X^* and M_{ZX}^* are constructed as follows.

The non-zero pattern of the $p \times p$ matrix S_X^* is identical across all simulations and is similar to the one adopted by Wytock & Kolter (2013): the graph over X is a chain of p variables in which one link out of five has been removed. The non-diagonal entries of S_X^* are such that $S_{Xij}^* \neq 0$, if and only if $i = j + 1$ and $i \not\equiv 0 \pmod 5$. The non-diagonal, non-zero entries are drawn from a uniform distribution: $\mathcal{U}([-1, -0.3] \cup [0.3, 1])$. The diagonal entries are drawn from $\mathcal{U}([0.5, 1.5])$. Figure 1 **a), b)** shows the precision matrix for both

X and H. S_X^* is shown in the bottom-left of each plot.

As stated in the main paper, the rank/sparsity of L_X^* is described by a single integer, d_H. Specifically, we assume that p is an integer of the form $p = 2^k$ and pick $d_H \in \{0, 1, \ldots, k\}$. Then, for a fixed value of d_H, M_H^* and M_{ZX}^* are random matrices constructed so that: a) there are exactly 2^{d_H} confounders, *i.e.* the rank of L_X^* is 2^{d_H}; b) each of the 2^{d_H} confounders impacts exactly $p/2^{d_H}$ outputs; c) each output is connected to exactly one latent variable. The latent variables are uncorrelated so M_H^* is a diagonal matrix with diagonal entries such that $(M_H^*)_{ii} \sim \mathcal{U}(0.3, 1)$. The non-zero entries of M_{XH}^* are drawn from $\mathcal{U}([-1.5, -0.5] \cup [0.5, 1.5])$. Finally, since drawing effect sizes from these distributions does not guarantee the positive-definitiveness of $\begin{pmatrix} S_X^* & M_{XH}^* \\ {M_{XH}^*}^T & M_H^* \end{pmatrix}$, we add a multiple of the identity matrix to it (ηI_{p+h}, say) and chose η so that the smallest eigenvalue be 0.05. In Figure 1 **a), b)** we give two examples for $p = 2^5 = 32$ and $d_H = 2$ and $d_H = 5$.

Likewise, d_Z accounts for the structure of M_{ZX}^*. Here again, we assume $p = 2^k$ and pick $d_Z \in \{0, 1, \ldots, k\}$. Then, M_{ZX}^* is designed to satisfy: a) $rank(M_{ZX}^*) = 2^{d_Z}$; b) each row/column of M_{ZX}^* has exactly $p/2^{d_Z}$ non-zero entries. The entries of M_{ZX}^* are drawn from $\mathcal{U}([-1.5, -0.5] \cup [0.5, 1.5])$. In Figure 1 **c), d)** we show two examples for $d_Z = 2$ and $d_Z = 5$. Note that, as stipulated earlier, the number of inputs Z remains constant and equal to the number of outputs: one the rank and incoherence of M_{ZX}^* change, not its dimensions.

4.2 Additional results

In the main paper, our main concern was to illustrate how methods differ in terms of identifiability and consistency. Setting p and m to a relatively small value (32) made it possible to capture most scenarios with only 16 graphical structures. Here, we are interested

a)

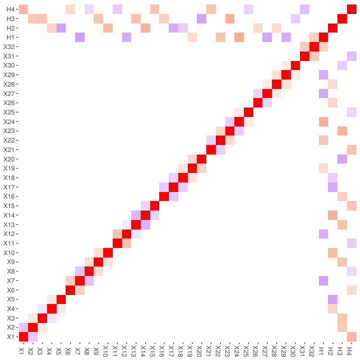

$d_H = 2.$

b)

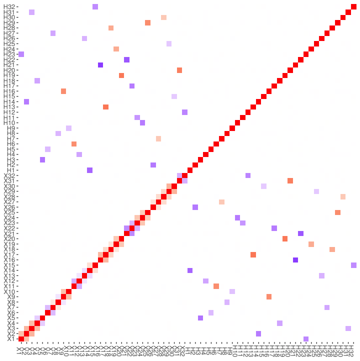

$d_H = 5.$

c)

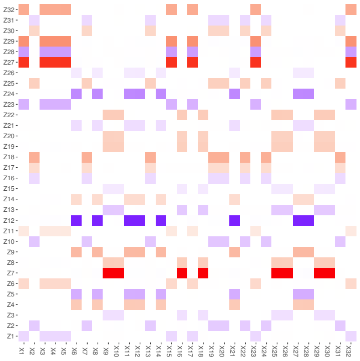

M^*_{ZX} for $d_Z = 2.$

d)

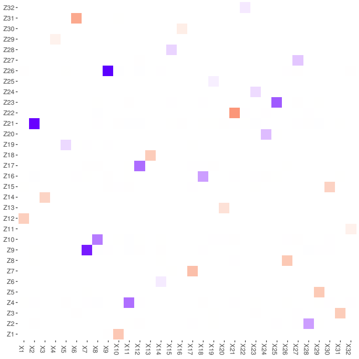

M^*_{ZX} for $d_Z = 5.$

Figure 1: Example of matrices used in our simulations, $p = 32$. In the top row, precision matrices over X and H for two values of d_H. In the bottom row, M^*_{ZX}, the matrix encoding the connection between inputs and outputs for two values of d_Z. The colour blue (resp. red) indicates a negative (resp. positive) entry. **a)** $d_H = 2$, there are four confounders acting on many outputs. **b)** $d_H = 5$, there are 32 latent variables, each acting on a single output. **c)** $d_Z = 2$, the 32 inputs are connected to the outputs via a low-rank matrix of rank 4. **d)** $d_Z = 5$: the 32 inputs are in a one-to-one correspondence with the outputs.

in simulating from a setting which is closer to the application considered in the next section. We set $p = 2^7 = 128$ but keep $n = 3000$. We consider four designs, repeated 20 times each: $(d_Z, d_H) \in \{4, 6\} \times \{3, 7\}$. $d_Z \in \{4, 6\}$ implies that the inputs act either in a low-rank or in a sparse, non-trivial, fashion. $d_H \in \{3, 7\}$ means that there is either low-rank confounding or no confounding at all.

Figure 2 shows the results obtained by selecting γ with perfect knowledge. When there is no confounding ($d_H = 7$), the precision/recall curves are similar to the ones obtained previously: both LR+S and LSCGGM achieve a high AUC. However, when $d_H = 3$, the AUCs of LR+S and LSCGGM are better than the ones shown in the main paper. The number of latent variables is identical in both cases but p is larger, which increases the incoherence between sparse and latent components.

From a practical perspective, one of the challenges of regularised maximum-likelihood estimators concerns the choice of tuning parameters. Since such estimators perform inference and model selection simultaneously, they can be used for a wide variety of tasks. In some cases, one might seek to maximise predictive power and not give much attention to the variables entering the model. In another context, it might be the opposite: the estimated effect sizes are discarded and only the variables being selected are of importance. These two problems are related, but what constitutes an "appropriate" value of the tuning parameters is very much application dependent.

Here, we selected γ using five-fold cross-validation, which is a simple and popular approach. Alternatives could be cross-validation with the "one standard-deviation rule" (Hastie et al. 2009) or model selection with the Extended Bayesian Information Criterion (Foygel & Drton 2010). In our application, we showed how one can select λ and γ for an application where some control over the number of falsely discovered edges is expected.

As observed in the main paper and other reports, neither LR+S nor LSCGGM are very

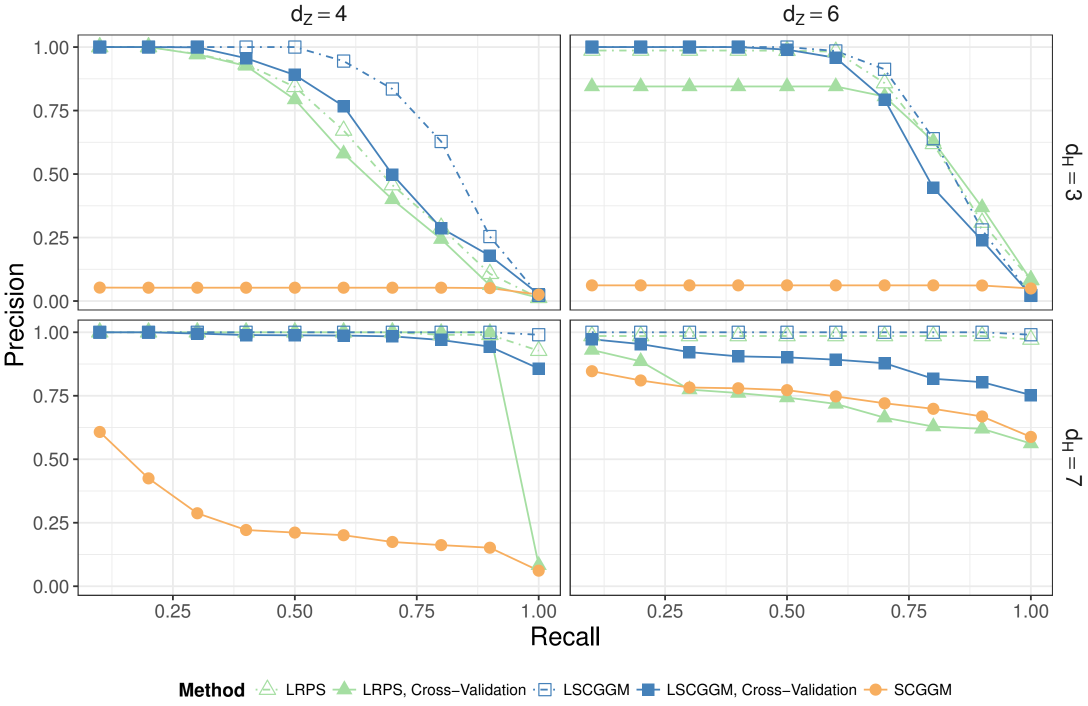

Figure 2: Comparison of LSCGGM to LR+S and SCGGM. From left to right (resp. top to bottom) d_Z (resp. d_H) takes values in $\{4, 6\}$ (resp. $\{3, 7\}$). In the bottom row, there is no confounding; in the top row there are eight latent variables. Likewise, on the left-hand side, inputs affect the outputs through a low-rank, diffuse, mechanism (the rank of K^*_{ZX} is 16). On the right-hand side, each input acts on two outputs (K^*_{ZX} is sparse). Settings: $p = 128, n = 3000$. For each dataset, the value of the tuning parameter γ is chosen either: a) so as to maximise the AUC (dashed lines, hollow markers) or b) using five-fold cross-validation (solid lines).

sensitive to the value of γ (Yuan 2012). Consequently, we used only ten values geometrically spaced within in the interval $[0.05, 0.8]$ – although four or five values would be enough for most applications.

As before, LSCGGM fares at least as well as LR+S. We believe that the relative insensitivity of LSCGGM to γ contributes to this result: the acceptable range is larger and it is easier to pick a value within this optimal interval. Finally, note that when there is no confounding and γ is selected with cross-validation (Figure 2, $d_H = 7, d_Z = 6$), the apparent advantage of low-rank plus sparse decompositions over a method such as SCGGM is reduced. The path chosen by cross-validation corresponds to a value of γ which is smaller than the one leading to the optimal AUC.

Finally, we show additional results for the estimation of L^* and S^*_{ZX}. In Figure 3, we plot the relative distance of the estimates to the *true* L^*, as a function of the recall of $\hat{S}_X$ (which is a proxy for λ_n). Figure 4, displays the precision recall curves obtained for recovery of the sparse matrix S^*_{ZX}. Whenever $d_Z = 6$, it is assumed that $L^*_{ZX} = 0$ and whenever $d_Z = 4$ it is assumed that $S^*_{ZX} = 0$ since the inputs do not act on the outputs in a sparse fashion at all. Likewise, for $d_H = 7$, we assume that $L^*_X = 0$.

5 Application: Supplementary Materials for Section 6

5.1 Data Preparation

The Avon Longitudinal Study of Parents and Children (ALSPAC) is a cohort study of children born in the county of Avon (United Kingdom) between 1991 and 1992. For obvious privacy reasons this dataset is not publicly available but the data can be requested by submitting a research proposal at the following address: `https://proposals.epi.bristol.`

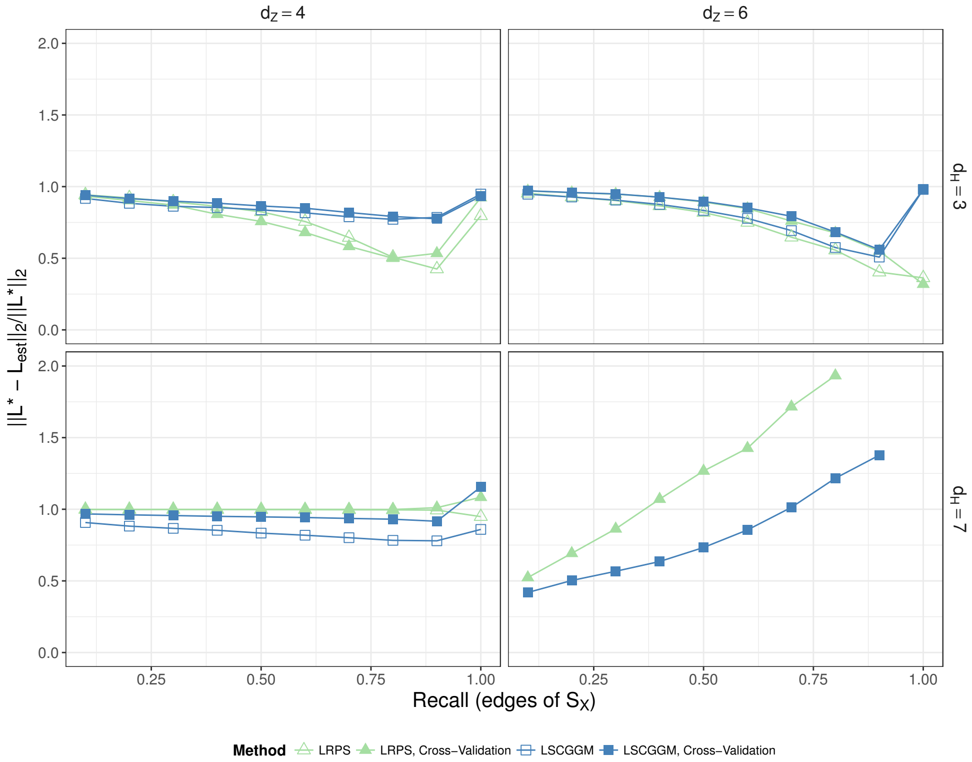

Figure 3: Relative distance between $\hat{L}$ and L^*, as measured by the spectral norm, for various values of d_H and d_Z. In the bottom right panel, the distance for non-cross validated estimates is not shown because it is too high on the y-axis. $p = 128, n = 3000$.

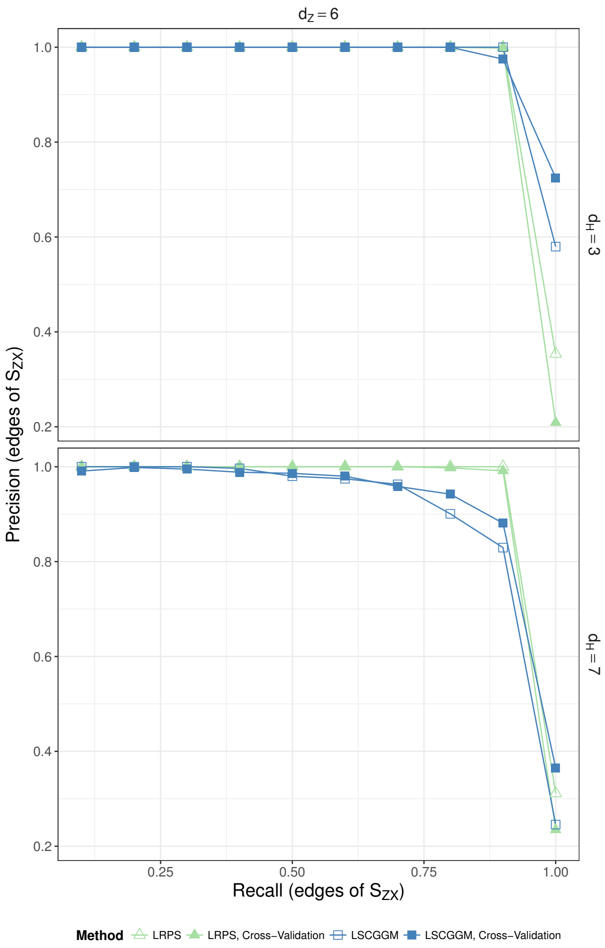

Figure 4: Precision vs. recall curves for the recovery of S^*_{ZX}. $p = 128, n = 3000$. Note that for the hollow shapes, the value of the tuning parameter γ was chosen so as to maximise the AUC of the precision vs. recall curve of S^*_X, *not* S^*_{ZX}. This ensures consistency across plots and makes comparisons easier.

`ac.uk/`.

In the dataset at our disposal, we identified around sixty metabolic traits of interest and kept the variables for which a sample size of 2,000 or more was available in both cohorts. 39 traits passed this filter and are listed in the table below. All traits were treated as continuous variables, quantile normalised and standardised.

For every sample, hundreds of thousands of genetic markers are measured. We therefore screened genetic variants in order to include only those markers that have a good predictive power for at least one of the 39 traits. This was achieved as follows. For every of the 39 metabolic traits, we performed independent univariate linear regressions of the trait on each of the genetic variants. Thus, for each trait, we obtained hundreds of thousands of p-values that we corrected for multiple testing using a Benjamini-Hochberg procedure. All variants that were significant at a False Discovery Rate of 10% were selected. In each cohort, a few hundred markers were significant at this FDR level but many of them were strongly correlated due to linkage disequilibrium (variants that are next to each other in the genome are typically very strongly correlated but often tag a single causal variant.) We selected variants so that their Pearson correlation be at most 0.2. 133 and 44 variants passed this filter in the child and mother cohorts respectively.

In conclusion, we obtained two independent datasets, one per cohort:

- Child Cohort: Sample Size $= 5,242$, $p = |X| = 39$, Number of inputs: $|Z| = m = 133$;
- Mother Cohort: Sample Size $= 2,770$, $p = |X| = 39$, Number of inputs: $|Z| = m = 44$.

5.2 Enrichment Statistic

In order to compute the enrichment statistic referred to in the main paper, we use *ChEBI: an ontology of small chemical entities of biological interest* Hastings et al. (2012).

First, we manually matched all 39 nodes to their ChEBI IDs. To each node we associate a set of ChEBI IDs consisting of its own ID, "related" terms' IDs and it's parents IDs (related terms and parents are defined by the ChEBI ontology). Then, we annotate the edges of each graphical structure by considering the intersection of its endpoints' annotations. For example, if node A is annotated by {ChEBI:001, ChEBI:002, ChEBI:003} and node B is annotated by {ChEBI:003, ChEBI:004, ChEBI:005}, an edge between A and B will be annotated by the set {ChEBI:003}.

For any given graph $\mathcal{G}$, let $\mathcal{T} = \{T_1, T_2, \ldots, \}$ denote the set of all the terms T_i that annotate at least two nodes. Then, for a given T_i, we write :

- $N(T_i)$ for the number of nodes annotated by term T_i;
- $E(T_i)$ for the number of edges annotated by term T_i.

We define the quantity S_i as follows:

$$S_i := \frac{E(T_i)}{N(T_i)(N(T_i) - 1))} \frac{N_n(N_n - 1)}{N_e},$$

where N_n is the number of nodes of $\mathcal{G}$ and N_e is the number of edges. Our enrichment statistic is then computed by taking the average of S_i over $\mathcal{T}$:

$$\mathcal{S} := \frac{1}{|\mathcal{T}|} \sum_i S_i.$$

An Erdős-Rényi random graph with the same nodes and the same number of edges as $\mathcal{G}$ has an expected statistic of 1. In practice, we compute the p-value associated to this metric by permuting the labels of $\mathcal{G}$. Thus, our null distribution is defined by a graph with the *same* topology as $\mathcal{G}$: vertex degrees, etc... are preserved.

5.3 Results for SCGGM

In Section 6 of the main paper, we applied our method (LSCGGM) and the Low-Rank plus Sparse (LR+S) estimator to the metabolite dataset described above. It was mentioned that the Sparse Conditional Gaussian Graphical Model (SCGGM) method arises as a special case of LSCGGM by setting the tuning parameter γ close to 0, so that the performance of SCGGM can be read off the plots by looking at the performance of LSCGGM when $\gamma = 0.02$. For completeness, we report here the results obtained by applying SCGGM to the Children and Mother datasets.

In Figure 5, the graphs returned by SCGGM after application of stability selection are plotted. The graph for the Children cohort has 16 edges, the graph for the Mother cohort has 11 edges. Their Jaccard similarity is 0.5.

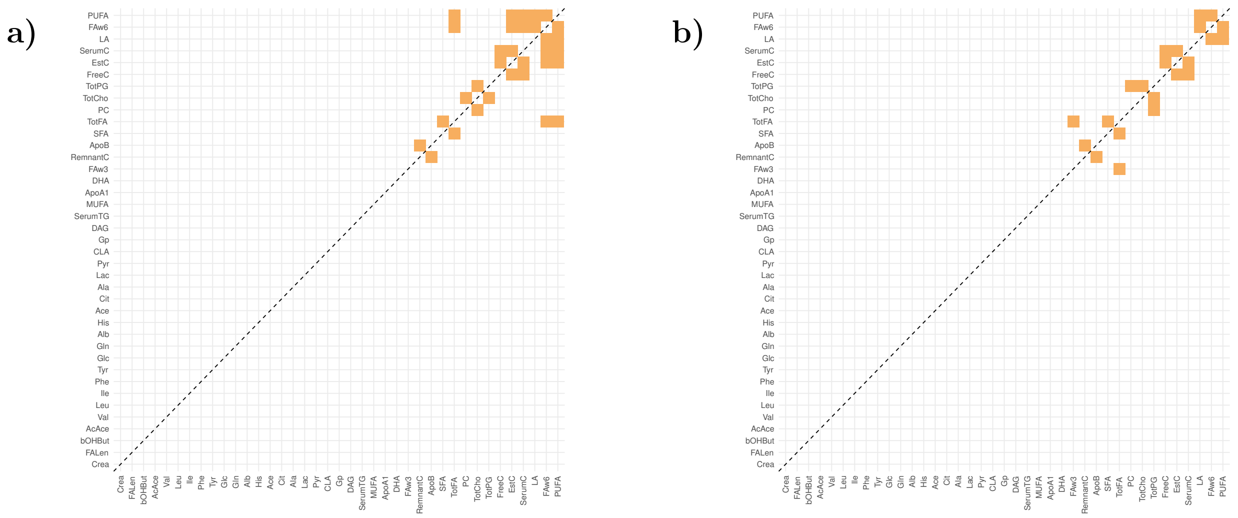

Figure 5: Estimates returned by SCGGM in the Children and Mother cohorts. Pointwise stability selection was applied as described in the main paper (we require $E(V) \leq 1$). (a) Children cohort. Tuning parameter $\lambda = 0.213$. Threshold on inclusion probabilities $\tau = 0.9$. (b) Mother cohort. Tuning parameter $\lambda = 0.217$. Threshold on inclusion probabilities $\tau = 0.88$.

In order to show the difference between methods that model latent variables (LSCGGM and LR+S) and SCGGM, we plot the solution path of SCGGM in Figure 6. Here, SCGGM was applied to the Children cohort and only edges with a selection probability greater than 50% after stability selection are shown.

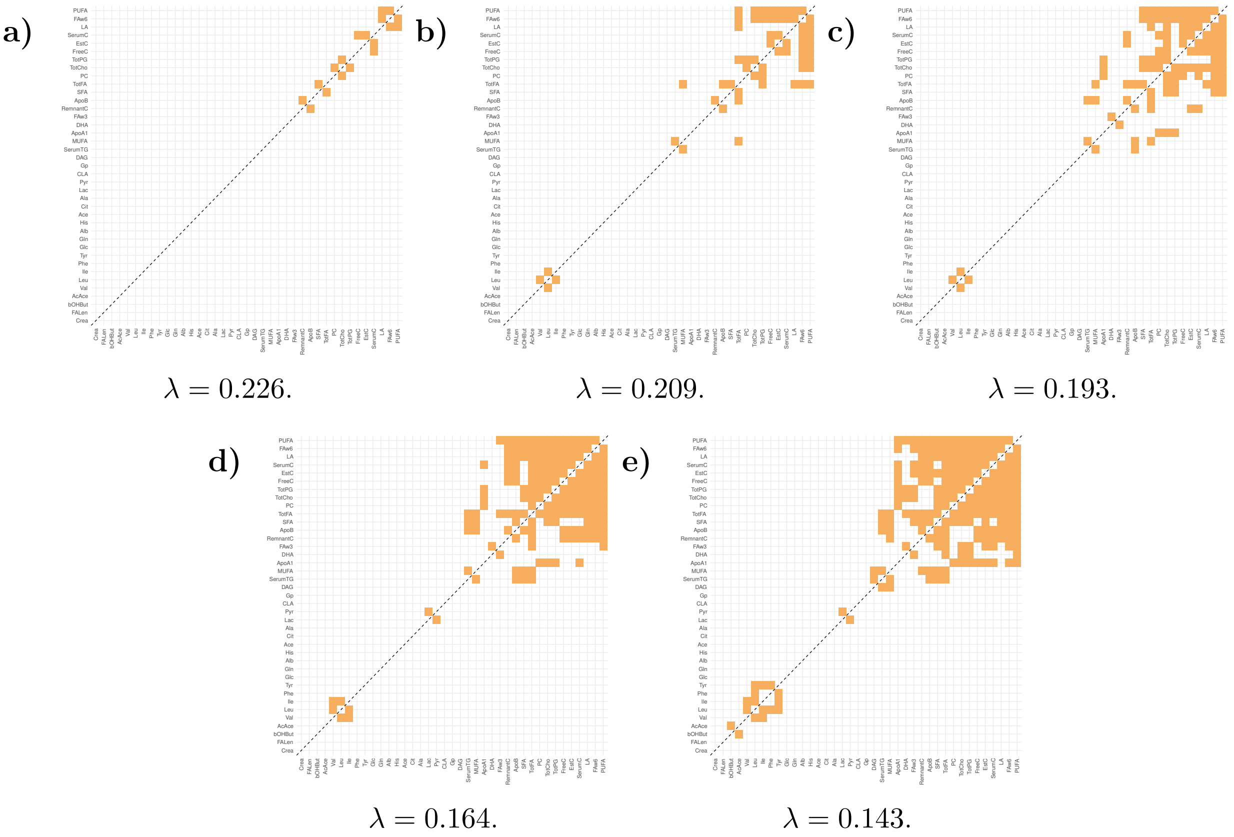

Figure 6: Solution path of SCGGM in the children cohort. For each value of the tuning parameter λ, complementary pairs stability selection was applied and edges with inclusion probabilities of 50% or less discarded.

For comparison, we plot in Figure 7 the solution path of our approach (LSCGGM) for the selected value of γ (0.81).

a)

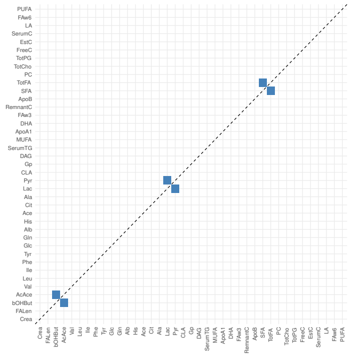

$\lambda = 0.027.$

b)

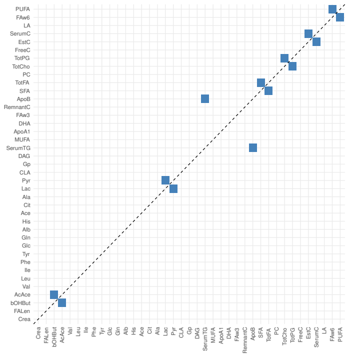

$\lambda = 0.018.$

c)

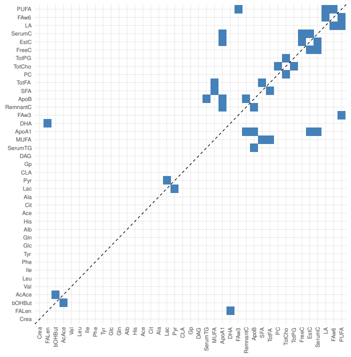

$\lambda = 0.010.$

d)

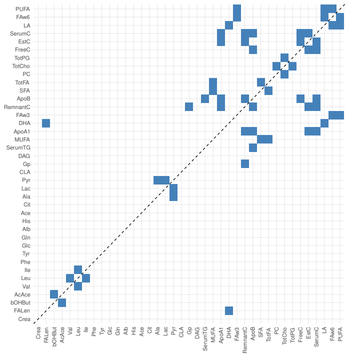

$\lambda = 0.0085.$

Figure 7: Solution path of LSCGGM in the children cohort, for $\gamma = 0.81$. For each value of the tuning parameter λ, complementary pairs stability selection was applied and edges with inclusion probabilities of 50% or less discarded.

5.4 Supplementary Figures

This section contains results complementing those reported in the main paper.

Figure 8 displays the Jaccard similarities of the edges sets across methods and cohorts, as a function of γ: **a)** Method: LR+S. Cohort: Children; **b)** Method: LSCGGM. Cohort: Mothers; **c)** Method: LR+S, Cohort: Mothers; **d)** Similarity of the edges sets of LSCGGM and LR+S in the Mother dataset.

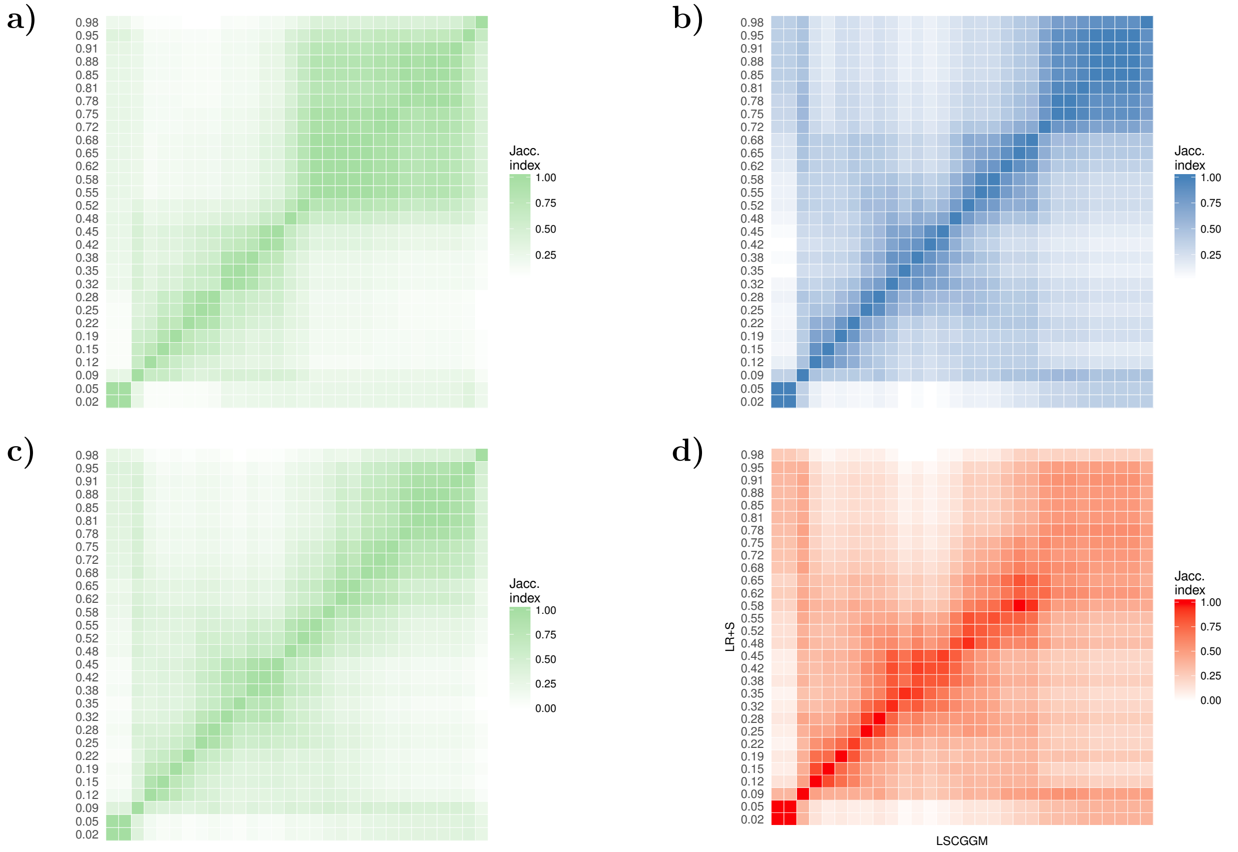

Figure 8: Sensitivity of LSCGGM and LR+S to the tuning parameter γ. For any two graphs, their similarity is the Jaccard Index of their edge sets. (a) Similarities between the edges sets of the graphs returned by LR+S in the Child cohort, as a function of γ (for 30 values of $\gamma \in (0.02, 0.98)$). (b) Similarities between the edges sets of the graphs returned by LSCGGM in the Mother cohort (c) Similarities between the edges sets of the graphs returned by LR+S in the Mother cohort (d) Similarities between the graphs returned by LSCGGM and LR+S in the Mother cohort.

Figure 9, shows the Jaccard similarity of a given method across cohorts, as a function of γ. This is an indicator of how well the results of a given method are replicated in an independent dataset. In the main paper, the values of the entries along the diagonal are reported, which makes comparing LSCGGM to LR+S easier. Figure 9 shows that the highest values tend to be reached along the diagonal, and not for very different values of γ.

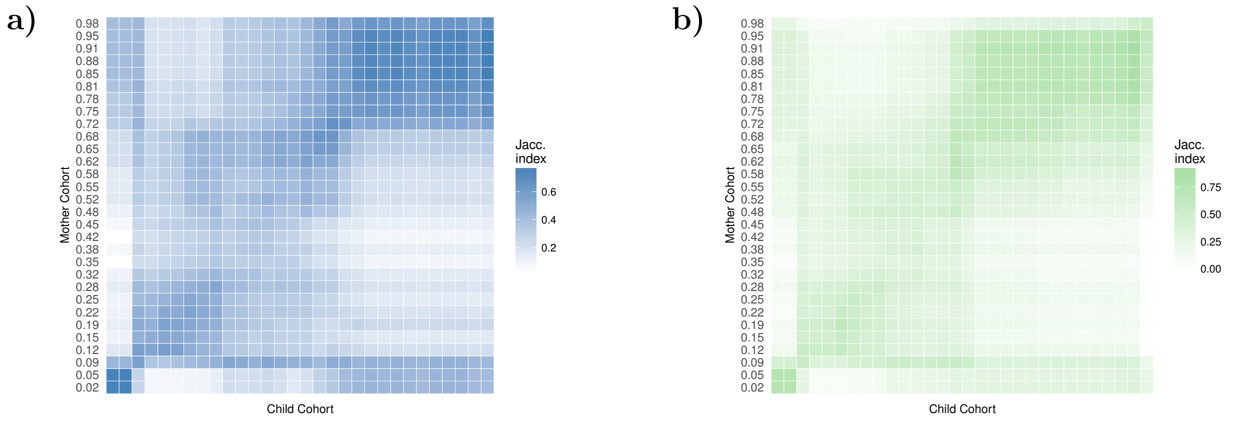

Figure 9: Comparing estimates across cohorts. For each possible pair (γ_1, γ_2) and each method, we plot the similarity between the estimate obtained in one cohort against the one obtained in the other. (a) LSCGGM Method. (b) LR+S.

Figure 10 reports the number of edges remaining after application of stability selection, for eahc method and in each cohort.

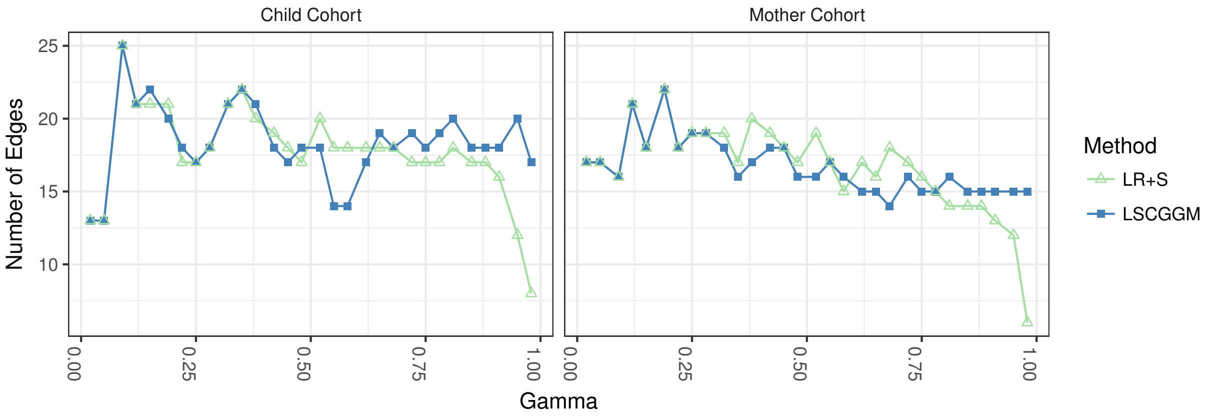

Figure 10: Number of edges returned as a function of γ. For every γ, in every cohort, we perform stability selection and ask that the expected number of falsely selected edges $E(V)$ be at most 1.

Finally, Figure 11 complements the plot of the main paper in which we report the value of our enrichment statistic. In this case, we show the empirical p-values obtained after 10^9 permutation of the annotations. In some cases, the empirical p-value is 0, which explains the saturation of the LSCGGM curve in the Mother cohort.

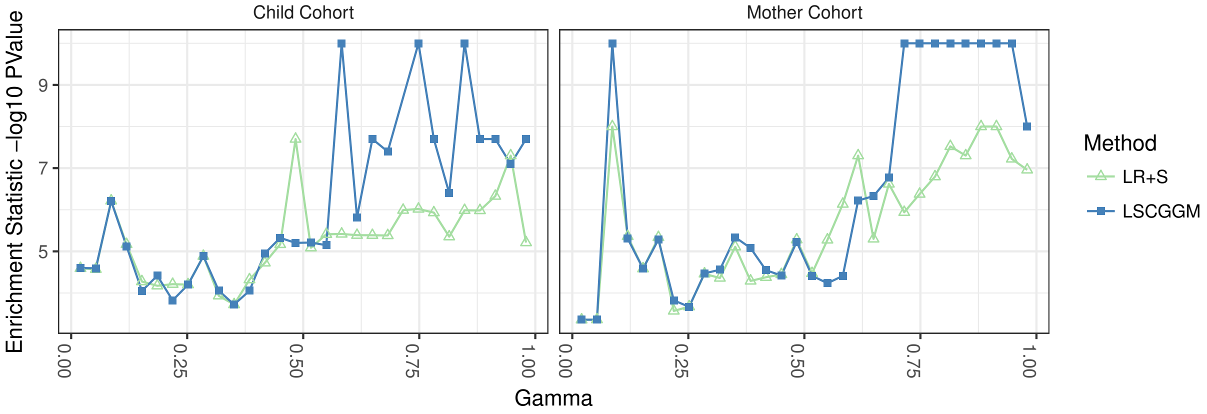

Figure 11: Empirical P-Values of the enrichment statistic $\mathcal{S}$ defined above. These P-Values are computed after 10^9 permutations. In some cases, the P-Value is 0: a graph with that value of $\mathcal{S}$ or higher was never seen after 1 billion permutations.

Variable	Description
serumc	serum total cholesterol (mmol/l)
remnantc	remnant cholesterol (non-hdl non-ldl -cholesterol) (mmol/l)
estc	esterified cholesterol (mmol/l)
freec	free cholesterol (mmol/l)
serumtg	serum total triglycerides (mmol/l)
dag	diacylglycerol (mmol/l)
totpg	total phosphoglycerides (mmol/l)
pc	phosphatidylcholine and other cholines (mmol/l)
totcho	total cholines (mmol/l)
apoa1	apolipoprotein a-i (g/l)
apob	apolipoprotein b (g/l)
totfa	total fatty acids (mmol/l)
falen	estimated description of fatty acid chain length not actual carbon number
dha	22:6-docosahexaenoic acid (mmol/l)
la	18:2-linoleic acid (mmol/l)
cla	conjugated linoleic acid (mmol/l)
faw3	omega-3 fatty acids (mmol/l)
faw6	omega-6 fatty acids (mmol/l)
pufa	polyunsaturated fatty acids (mmol/l)
mufa	monounsaturated fatty acids; 16:1 18:1 (mmol/l)
sfa	saturated fatty acids (mmol/l)
glc	glucose (mmol/l)
lac	lactate (mmol/l)
pyr	pyruvate (mmol/l)
cit	citrate (mmol/l)
ala	alanine (mmol/l)
gln	glutamine (mmol/l)
his	histidine (mmol/l)
ile	isoleucine (mmol/l)
leu	leucine (mmol/l)
val	valine (mmol/l)
phe	phenylalanine (mmol/l)
tyr	tyrosine (mmol/l)
ace	acetate (mmol/l)
acace	acetoacetate (mmol/l)
bohbut	3-hydroxybutyrate (mmol/l)
crea	creatinine (mmol/l)
alb	albumin (signal area)
gp	glycoprotein acetyls mainly a1-acid glycoprotein (mmol/l)

6 A Summary of our Notations

- m : number of variables we condition on. With our notations, $m = |Z|$.
- p : number of variables being modelled. With our notations, $p = |X|$.

- $||M||_2 = \sqrt{\text{largest eigenvalue of } M^T M}$: spectral norm of M.
- $||M||_\infty$: largest entry in magnitude of M.
- $||M||_1 = \sum_{i,j} |M_{ij}|$: sum of the absolute value of the entries of M.
- $||M||_* = \sum_i \sigma_i$: sum of the singular values of M.
- $g_\gamma(A, B) = \max(\frac{||A||_\infty}{\gamma}, ||B||_2)$.
- $\mathcal{A}$, the operator that adds two matrices.
- $\mathcal{A}^\dagger$: $\mathcal{A}^\dagger(A) = (A, A)$.
- $\mathcal{P}_T$: orthogonal projector onto the linear subspace T.
- $\rho(M_1, M_2) = \max_{||N||_2 \leq 1} ||(\mathcal{P}_{T_1} - \mathcal{P}_{T_1})(N)||_2$.
- $\mathcal{L}(r)$: variety of matrices of size $(m+p) \times p$ of rank at most r.
- $T(M)$, for M a matrix of rank r: the tangent space to $\mathcal{L}(r)$ at M.
- $\mathcal{S}(k)$: variety of matrices of size $(m+p) \times p$ with at most k non-entries.
- $\Omega(M)$, for M a matrix with k non-entries: tangent space to $\mathcal{S}(k)$ at M.
- $\xi(T(M)) = \max_{N \in T(M), ||N||_2 \leq 1} ||N||_\infty$.
- $\mu(\Omega(M)) = \max_{N \in \Omega(M), ||N||_\infty \leq 1} ||N||_2$.
- $\mathcal{I}^*_{\Sigma^n_Z}$: Fisher Information Matrix evaluated at the nominal parameters.
- $\alpha_\Omega \triangleq \min_{M \in \Omega, ||M||_\infty = 1} ||\mathcal{P}_\Omega \mathcal{I}^*_{\Sigma^n_Z} \mathcal{P}_\Omega(M)||_\infty$.

- $\delta_\Omega \triangleq \max_{M \in \Omega, ||M||_\infty = 1} ||\mathcal{P}_{\Omega^\perp} \mathcal{I}^*_{\Sigma^n_Z} \mathcal{P}_\Omega(M)||_\infty$.
- $\beta_\Omega \triangleq \max_{M \in \Omega, ||M||_2 = 1} ||\mathcal{I}^*_{\Sigma^n_Z}(M)||_2$.
- $\alpha_T \triangleq \min_{\rho(T,T')<\xi(T)/2} \min_{M \in T', ||M||_2 = 1} ||\mathcal{P}_{T'} \mathcal{I}^*_{\Sigma^n_Z} \mathcal{P}_{T'}(M)||_2$.
- $\delta_T \triangleq \max_{\rho(T,T')<\xi(T)/2} \max_{M \in T', ||M||_2 = 1} ||\mathcal{P}_{T'^\perp} \mathcal{I}^*_{\Sigma^n_Z} \mathcal{P}_{T'}(M)||_2$
- $\beta_T \triangleq \max_{\rho(T,T')<\xi(T)/2} \max_{M \in T', ||M||_\infty = 1} ||\mathcal{I}^*_{\Sigma^n_Z}(M)||_\infty$.
- $\alpha \triangleq \min(\alpha_\Omega, \alpha_T)$.
- $\beta \triangleq \max(\beta_\Omega, \beta_T)$.
- $\delta \triangleq \max(\delta_\Omega, \delta_T)$.
- ν: a number in $(0, \frac{1}{2}]$ such that $\frac{\delta}{\alpha} \leq 1 - 2\nu$.
- $w = \max(1, \frac{1}{\gamma})$.
- $D = \max(1, \frac{\nu\alpha}{3\beta(2\nu)})$.

References

Bach, F. (2008), 'Consistency of trace norm minimization', *J. Mach. Learn. Res.* **8**, 1019–1048.

Boyd, S. (2011), 'Alternating direction method of multipliers', *Proceedings of the 51st IEEE Conference on Decision and Control* .

Boyd, S., Parikh, N., Chu, E., Peleato, B. & Eckstein5, J. (2010), 'Distributed optimization and statistical learning via the alternating direction method of multipliers', *Foundations and Trends in Machine Learning* **3**(1), 1–122.

Chandrasekaran, V., Parrilo, P. A. & Willsky, A. S. (2012), 'Latent variable graphical model selection via convex optimization', *The Annals of Statistics* **40**(4), 1935–1967.

Chandrasekaran, V., Sanghavi, S., Parrilo, P. A. & Willsky, A. S. (2009), 'Rank-sparsity incoherence for matrix decomposition', *SIAM Journal on Optimization* **21**(2), 572–596.

Chen, C., He, B., Ye, Y. & Yuan, X. (2016), 'The direct extension of admm for multi-block convex minimization problems is not necessarily convergent', *Math. Program.* **155**(1-2), 57–79.

Combettes, P. L. & Pesquet, J.-C. (2009), Proximal splitting methods in signal processing, *in* 'Fixed-Point Algorithms for Inverse Problems in Science and Engineering', chapter 10, pp. 185–212.

Davidson, K. R. & Szarek, S. J. (2001), Handbook of the geometry of banach spaces, *in* 'Handbook of the Geometry of Banach Spaces'.

Foygel, R. & Drton, M. (2010), Extended bayesian information criteria for gaussian graphical models, *in* J. Lafferty, C. Williams, J. Shawe-taylor, R. Zemel & A. Culotta, eds, 'Advances in Neural Information Processing Systems 23', pp. 604–612.

Goldstein, T. & Osher, S. (2009), 'The split bregman method for l1-regularized problems', *SIAM Journal on Imaging Sciences* **2**(2), 323–343.

Hastie, T., Tibshirani, R. & Friedman, J. (2009), *The Elements of Statistical Learning*, Springer New York.

Hastings, J. et al. (2012), 'The ChEBI reference database and ontology for biologically relevant chemistry: Enhancements for 2013', *Nucleic Acids Research* **41**(D1), D456–D463.

Lin, T., Ma, S. & Zhang, S. (2016), 'Iteration complexity analysis of multi-block admm for a family of convex minimization without strong convexity', *Journal of Scientific Computing* **69**(1), 52–81.

Löfberg, J. (2004), Yalmip : A toolbox for modeling and optimization in matlab, *in* 'Proceedings of the CACSD Conference', pp. 284 – 289.

Ma, S., Xue, L. & Zou, H. (2013), 'Alternating direction methods for latent variable gaussian graphical model selection', *Neural Computation* **25**(8), 2172–2198.

Parikh, N. & Boyd, S. (2014), 'Proximal algorithms', *Foundations and Trends in Optimization* **1**(3), 127–239.

Ravikumar, P., Wainwright, M. J., Raskutti, G. & Yu, B. (2011), 'High-dimensional covariance estimation by minimizing l1-penalized log-determinant divergence', *Electronic Journal of Statistics* **5**.

Stewart, G. & Sun, J.-g. (1990), *Matrix Perturbation Theory*, Academic Press.

Tütüncü, R. H., Toh, K. C. & Todd, M. J. (2003), 'Solving semidefinite-quadratic-linear programs using sdpt3', *Mathematical Programming, Series B* **95**(2), 189–217.

Vandenberghe, L. & Boyd, S. (1996), 'Semidefinite programming', *SIAM Review* **38**(1), 49–95.

Wainwright, M. J. (2009), 'Sharp thresholds for high-dimensional and noisy sparsity recovery using l1-constrained quadratic programming (lasso)', *IEEE Transactions on Information Theory* **55**(5), 2183–2202.

Wang, C., Sun, D. & Toh, K.-C. (2010), 'Solving log-determinant optimization problems by a newton-cg primal proximal point algorithm', *SIAM Journal on Optimization* **20**(6), 2994–3013.

Wytock, M. & Kolter, J. Z. (2013), Sparse gaussian conditional random fields: Algorithms, theory, and application to energy forecasting, *in* 'Proceedings of the 2013 International Conference on Machine Learning', pp. 1265–1273.

Yang, L., Sun, D. & Toh, K.-C. (2015), 'Sdpnal+: a majorized semismooth newton-cg augmented lagrangian method for semidefinite programming with nonnegative constraints', *Mathematical Programming Computation* **7**(3), 331–366.

Ye, G.-B., Wang, Y. & Xie, X. (2011), 'Efficient latent variable graphical model selection via split bregman method', *Available on the arXiv at http://arxiv.org/pdf/1110.3076v1.pdf* .

Yin, J. & Li, H. (2011), 'A sparse conditional gaussian graphical model for analysis of genetical genomics data.', *The annals of applied statistics* **5**(4), 2630–2650.

Yuan, M. (2012), 'Discussion: Latent variable graphical model selection via convex optimization', *The Annals of Statistics* **40**(4), 1968–1972.
